# Fecal Aβ and tau aggregates are elevated in cognitive impairment and reveal peripheral proteopathic alterations

**DOI:** 10.1186/s13195-026-02188-0

**Published:** 2026-09-26

**Authors:** Pelin Özdüzenciler, Laura Müller, Esra Kara, Maria-Christina Weber, Marlene Pils, Fabian Rehn, Oliver Bannach, Gereon R. Fink, Dieter Willbold, Michael T. Barbe, Oezguer A. Onur, Gültekin Tamgüney

**Affiliations:** 1 Institut für Physikalische Biologie, Heinrich-Heine-Universität Düsseldorf, Düsseldorf, 40225 Germany; 2 Institute of Biological Information Processing, Structural Biochemistry (IBI-7), Forschungszentrum Jülich, Jülich, 52428 Germany; 3 Department of Neurology, Faculty of Medicine and University Hospital Cologne, University of Cologne, Köln, 50923 Germany; 4 attyloid GmbH, Düsseldorf, 40225 Germany; 5 Cognitive Neuroscience, Institute of Neuroscience and Medicine (INM-3), Jülich Research Centre, Jülich, 52428 Germany

**Keywords:** Alzheimer’s disease, Amyloid-β aggregates, Tau aggregates, Fecal biomarkers, Non-invasive diagnostics, Gut-brain axis, sFIDA

## Abstract

**Background:**

Aggregation of amyloid-β (Aβ) and tau is central to Alzheimer’s disease (AD) pathogenesis and forms the basis of established cerebrospinal fluid (CSF) and imaging biomarkers. However, increasing evidence suggests that AD involves systemic processes beyond the central nervous system. We previously demonstrated the presence of fecal Aβ aggregates. Here, we assessed the detectability of tau aggregates in human feces and examined the diagnostic value of combined fecal Aβ and tau quantification in cognitive impairment.

**Methods:**

Using surface-based fluorescence intensity distribution analysis (sFIDA), a single-particle assay selective for aggregated species, we quantified fecal Aβ and tau aggregates in individuals with cognitive impairment (dementia, MCI, SCD) and cognitively normal controls. Associations with CSF biomarkers were assessed, and classification performance was evaluated using cross-validated logistic regression models.

**Results:**

Tau aggregates were detectable in fecal samples. Both fecal Aβ and tau aggregate concentrations were significantly elevated in cognitively impaired individuals and remained independently associated with patient status after adjustment for age and sex. Fecal Aβ aggregates showed inverse associations with CSF tau biomarkers, particularly phosphorylated tau. In cross-validated models, fecal tau aggregates achieved stronger classification performance than fecal Aβ (AUC ~ 0.80 vs. ~ 0.68), and integration of fecal aggregates with demographic predictors improved discrimination to an AUC of 0.90.

**Conclusions:**

Detection of fecal tau aggregates extends previous observations on fecal Aβ and supports the concept that stool-based aggregate measurements capture systemic aspects of proteopathic biology associated with cognitive impairment. These findings suggest that fecal aggregate quantification may provide a minimally invasive biomarker approach that complements established CNS-based biomarkers and may be useful for patient stratification and monitoring in neurodegenerative disease.

**Supplementary Information:**

The online version contains supplementary material available at https://doi.org/10.1186/s13195-026-02188-0.

## Background

Alzheimer’s disease (AD) is the most common cause of dementia and represents a major global health burden [1]. Neuropathologically, AD is characterized by the accumulation of extracellular amyloid-β (Aβ) aggregates and intracellular tau pathology, leading to synaptic dysfunction, neuronal loss, and progressive cognitive decline [2–5]. Although these pathological processes originate in the central nervous system (CNS), increasing evidence suggests that protein aggregation associated with neurodegeneration may also be reflected in peripheral biological compartments. The introduction of biologically defined diagnostic frameworks, particularly the AT(N) classification system, has substantially refined the conceptualization of AD as a continuum defined by amyloid deposition (A), tau pathology (T), and neurodegeneration (N) [2, 6]. Established cerebrospinal fluid (CSF) and positron emission tomography (PET) biomarkers now enable in vivo detection of these processes with high accuracy [7–11]. However, CSF sampling remains invasive, and PET imaging is costly and not universally accessible, highlighting the need for minimally invasive biomarkers that capture core aspects of AD-related proteinopathy.

Protein misfolding and aggregation are central to AD pathogenesis. While fibrillar plaques and neurofibrillary tangles define the neuropathological hallmarks of the disease, increasing evidence indicates that smaller soluble Aβ and tau aggregates, including oligomeric species, may be particularly neurotoxic and mechanistically linked to synaptic dysfunction and disease progression [5, 12]. Aggregate dynamics are influenced not only by production but also by clearance mechanisms. In addition to glymphatic and blood-brain barrier-mediated pathways, peripheral organs, including the liver, have been implicated in Aβ clearance and systemic elimination [13–16]. These observations raise the possibility that central proteopathic processes may be reflected in peripheral compartments.

Converging experimental and clinical evidence supports bidirectional communication between the gastrointestinal system and the CNS, commonly referred to as the gut-brain axis, involving neural, immune, endocrine, and microbial signaling pathways [17]. Alterations in gut microbiota composition, intestinal permeability, and systemic inflammatory signaling have been reported in AD and in prodromal stages of the disease [18–20]. Microbial amyloids and metabolites may influence host protein aggregation and immune responses, supporting the concept of a gut-brain axis in neurodegeneration [21]. While several studies have examined microbiome composition in AD, comparatively little is known about intestinal or fecal Aβ and tau aggregates themselves. Initial reports demonstrated the presence of Aβ peptides in intestinal tissue and fecal material, and exploratory studies suggested increased fecal Aβ levels in AD [22–26]. However, these studies did not systematically assess aggregate species or relate intestinal findings to CSF-defined AT biomarker status.

We previously developed surface-based fluorescence intensity distribution analysis (sFIDA), a microscopy-based single-particle detection platform enabling sub-femtomolar quantification of protein aggregates while minimizing monomer interference through epitope-overlapping antibody design [27]. Using sFIDA, we previously demonstrated the feasibility of detecting Aβ aggregates in human feces in a proof-of-concept cohort and established the utility of sFIDA for quantifying Aβ and tau aggregates in CSF, where aggregate concentrations are associated with clinical diagnosis and biomarker-defined AD [22, 28, 29]. However, key questions remain unresolved: the reproducibility of fecal aggregate detection in a larger biomarker-characterized cohort, the detectability of fecal tau aggregates in human stool, and the relationship between intestinal aggregate signals and canonical CSF Aβ and tau biomarkers.

In the present study, we applied analytically validated sFIDA assays to quantify fecal Aβ and tau aggregates in a well-characterized cohort of patients with cognitive impairment and cognitively healthy controls. We examined associations with CSF Aβ_1–42_, total tau, phosphorylated tau, and AT classification, and evaluated the incremental diagnostic performance of fecal aggregates beyond demographic variables. By integrating intestinal aggregate quantification with established CSF biomarkers, we aimed to determine whether fecal protein aggregates represent a biologically informative and minimally invasive marker linked to central tau pathology.

## Methods

### Study cohort and diagnostic classification

Participants were recruited at the Department of Neurology, University Hospital Cologne. Individuals without neurological disease, subjective cognitive complaints, evidence of neuropsychological deficits, or impairment in activities of daily living (ADL) were classified as cognitively healthy controls (HC). Patients presenting with cognitive decline were evaluated by an interdisciplinary team with expertise in dementia diagnosis. Clinical diagnoses were established according to National Institute on Aging-Alzheimer’s Association (NIA-AA) criteria based on clinical evaluation and neuropsychological assessment [30]. Patients with cognitive impairment and preserved ADL were classified as having mild cognitive impairment (MCI), whereas those with impaired ADL were diagnosed with dementia. Individuals reporting subjective cognitive decline (SCD) but demonstrating normal performance on objective cognitive testing and preserved ADL were classified as having SCD. CSF and/or PET biomarker measurements were analyzed to determine AT biomarker status. If Functional Activities Questionnaire (FAQ) data were unavailable, functional status was determined based on structured clinical history and caregiver interview.

### Neuropsychological and functional assessments

Global cognitive performance was assessed using the DemTect screening instrument (score range 0–18). Scores > 12 were considered age-adjusted normal, 9–12 consistent with MCI, and < 9 indicative of dementia [31]. Depressive symptoms were evaluated using the German version of the Beck Depression Inventory II (BDI-II; score range 0–63), with established severity categories (minimal, mild, moderate, severe) [32]. Subjective memory complaints were assessed using the Memory Assessment Clinic Questionnaire (MAC-Q, score range 7–35); scores > 24 were considered clinically relevant [33]. Functional impairment was determined using the FAQ (score range 0–30), with scores > 8 indicative of clinically relevant impairment and required for a diagnosis of dementia [34].

### CSF and PET biomarker assessment

To determine AD etiology, biomarker-based diagnostics were performed [2]. Biomarker classification followed the NIA-AA AT(N) framework. Amyloid positivity (A+) was defined by abnormal amyloid PET or CSF evidence of amyloid pathology based on validated assay-specific thresholds for Aβ_1–42_, the Aβ_1–42_/Aβ_1–40_ ratio and/or a phosphorylated tau181 (p-tau181)/Aβ_1–42_ ratio. Tau positivity (T+) was defined by elevated CSF p-tau181 and/or positive tau PET. Total tau was analyzed separately as a marker of neuronal injury and neurodegeneration (N). Prior to 2022, CSF biomarkers were quantified using commercially available enzyme-linked immunosorbent assays (ELISA; Euroimmun AG; Fujirebio Europe N.V.). Amyloid positivity was defined as Aβ_1–42_ < 630 pg/mL or Aβ_1–42_/Aβ_1–40_ ratio ≤ 0.095. Elevated total tau was defined as > 452 pg/mL, and p-tau181 > 61 pg/mL was considered pathological. From 2022 onward, biomarkers were measured using automated electrochemiluminescence immunoassays (Elecsys, Roche Diagnostics) on a cobas e 801 analyzer. With these assays, amyloid positivity was defined as Aβ_1–42_ ≤ 1030 pg/mL, total tau > 300 pg/mL, p-tau181 > 27 pg/mL, and/or a p-tau181/Aβ_1–42_ ratio > 0.0230.

### Assessment of gastrointestinal symptoms

Gastrointestinal symptoms were assessed using the Cleveland Clinic Constipation Scoring System (CCCSS). The CCCSS is an eight-item, patient-reported questionnaire evaluating bowel movement frequency, difficulty of evacuation, sensation of incomplete evacuation, time spent per attempt, abdominal pain, use of assistance or laxatives, unsuccessful attempts, and duration of symptoms. Total scores range from 0 to 30, with higher scores indicating more severe constipation. The CCCSS was administered at the time of biological sample collection. For statistical analyses, the total score was treated as a continuous variable [35].

### Fecal sample collection and homogenization

Fecal samples were collected between September 2020 and November 2024 in polypropylene sample tubes with a screwcap-integrated spoon (megro, Wesel, Germany) and a paper-based collection aid (Med Auxil analysis aids, Seesen, Germany). Samples were aliquoted into polypropylene protein low-binding tubes (Sarstedt, Nümbrecht, Germany) for storage at − 80 °C. For homogenization, 17-mg samples were transferred to Simplix polypropylene tubes (Gaudlitz, Coburg, Germany) containing 1300 µL of homogenization buffer (20 mM Tris, 250 mM NaCl, pH 8.3; AppliChem, Darmstadt, Germany) supplemented with 1× cOmplete EDTA-free protease inhibitor and 1× PhosStop phosphatase inhibitor (Roche, Basel, Switzerland), 5% glycerol, 0.05% NaN_3_ (AppliChem), and 1% bovine serum albumin (BSA, AppliChem). BSA was omitted from the buffer used for measurements of tau aggregates. Samples were homogenized using a vortex mixer for 10 min at room temperature, then transferred to 2 mL polypropylene low-binding tubes (Eppendorf, Hamburg, Germany) and centrifuged at 3000 × g for 5 min at 4 °C. The supernatants were subsequently transferred to 1.5 mL polypropylene low-binding tubes and stored at − 80 °C until further use. Homogenates were diluted 1:5 in sample buffer containing phosphate-buffered saline (PBS, Sigma-Aldrich), 0.05% Tween (AppliChem), 0.095% NaN_3_, and 0.5% BSA for measuring Aβ aggregates, or in LowCross-Buffer (Candor, Wangen, Germany) for measuring tau aggregates.

### sFIDA assay

The principle of sFIDA was reported previously [22, 28, 36, 37]. To capture Aβ or tau aggregates, a 384-well glass-bottom microplate (SensoPlate plus, Greiner Bio-One, Frickenhausen, Germany) was incubated with 2.5 µg/mL of monoclonal IC16 anti-Aβ antibody directed against amino acids 2 − 8 (kindly provided by Carsten Korth, Universitätsklinikum Düsseldorf, Germany), or Tau-12 anti-Tau antibody directed against amino acids 6 − 18 (Biolegend, San Diego, USA) in 0.1 M NaHCO_3_ overnight at 4 °C [38]. Subsequently, the plates were washed five times with Tris-buffered saline (TBS; Serva, Duisburg, Germany) with 0.05% Tween 20 (TBST), and then five times with TBS. To block free binding sites, the wells were incubated for 1.5 h at room temperature with 0.5% BSA in TBS with 0.03% ProClin (TBS-ProClin; Sigma-Aldrich, Missouri, USA) for the detection of Aβ aggregates or with Blocking Solution (Candor, Wangen, Germany) for the detection of tau aggregates. Next, the wells underwent five washes with TBST and TBS. Silica nanoparticles (SiNaPs) coated with Aβ_1–15_ or full-length tau functioned as a standard for protein quantification and were prepared as previously described and diluted in sample buffer or LowCross-Buffer, respectively [22, 28, 39]. Four replicates of 20 µL of each SiNaP dilution or of 1:5 diluted samples were added to the wells and incubated for 2 h at room temperature. Subsequently, the plate was washed five times with TBS. To detect Aβ and tau aggregates, the wells were incubated for 1 h with fluorescent IC16-CF633 and IC16-CF488A antibodies each at 0.625 µg/mL in TBST containing 0.1% BSA, or Tau-12-CF633 and Tau-12-CF488A antibodies, each at 0.156 µg/mL in TBS, respectively. Finally, the plate was washed five times with TBS and the buffer exchanged to TBS-ProClin for measurement. An automated microtiter plate washer (405 LS Microplate Washer, BioTek, VT, USA) carried out all washing steps.

Images were captured using an IN Cell Analyzer 6500HS (GE Healthcare, Freiburg, Germany) with a 40× objective through two-channel confocal fluorescence imaging. The first channel was excited at 642 nm, using an emission filter at 682.5/59 nm, with an exposure time set to 4 s. The second channel utilized an excitation wavelength of 488 nm, with an emission filter at 524.5/48 nm and an exposure time of 5 s. A total number of 25 images per well, each measuring 2040 × 2040 pixels, were captured for each channel. sFIDAta version 2.2.7 was utilized for the automated detection and elimination of images with artifacts, and for quantifying colocalized pixels in both channels (sFIDA readout) [40]. To minimize background, a cutoff was established as the grey value at which 0.001% of all pixels in images of the buffer control were positive. Only pixels with grey values above the cutoff were analysed. The limit of detection (LOD) was defined as the sFIDA readout of the buffer control plus two standard deviations. A calibration curve was fitted using sFIDA readouts of dilutions of SiNaPs of known concentration and second-order polynomial regression. This calibration curve was used to calculate a concentration for each sFIDA readout. Because biological fecal aggregates may differ from the SiNaP standards in size, morphology, epitope density, and antibody accessibility, the resulting fM values represent SiNaP-equivalent concentrations rather than absolute molar concentrations of native aggregate particles. The mean value and the standard deviation of the sFIDA readouts and the coefficient of variation (CV%) for each sample were calculated by the sFIDAta software based on the four replicates. Representative images shown in Additional file 1: Fig. S1 were selected according to predefined quantitative sFIDA readout positions and are provided only to illustrate the detected dual-channel signals; statistical analyses were based on automated quantification of all acquired images rather than visual assessment of selected fields.

### Statistical analysis

Statistical analyses were performed using GraphPad Prism (version 11) and RStudio (version 2026.01.1). Two-sided *p*-values < 0.05 were considered statistically significant. Continuous variables are presented as mean ± standard deviation (SD) or median with interquartile range (IQR), as appropriate. Group differences in fecal aggregate concentrations between cognitively healthy controls (HC) and patients with cognitive impairment were assessed using two-sided Mann-Whitney U tests due to non-normal distribution of aggregate concentrations. Effect sizes for pairwise comparisons were quantified using Cliff’s δ. Global comparisons across CSF-defined AT biomarker strata were performed using Kruskal-Wallis tests. To address the age imbalance between cognitively healthy controls and patients, the primary patient-control comparison was additionally presented using an age-restricted control subset aged ≥ 60 years. For regression and visualization analyses, fecal aggregate concentrations were log_10_-transformed after addition of a small constant (0.01 fM) to zero values. Associations between fecal aggregates and CSF biomarkers within the patient cohort were evaluated using linear regression, and coefficients of determination (R^2^) are reported. Multivariable linear regression models were constructed to assess independent associations, including age, sex, and diagnostic category (AD vs. non-AD) as covariates. Because years of education differed numerically between groups, additional sensitivity analyses were performed including education as a covariate in the patient-control regression models. Regression diagnostics were assessed for all multivariable linear and logistic regression models. For linear regression models, residual plots were inspected to evaluate approximate linearity, residual structure, and model fit. Influential observations were assessed using Cook’s distance. For logistic regression models, model specification and goodness-of-fit were evaluated using calibration/goodness-of-fit diagnostics, and influential observations were examined. Multicollinearity was assessed using variance inflation factors (VIFs). No relevant multicollinearity was detected in the multivariable models, with all VIF values below 1.5. For models evaluating patient-control differences, regression coefficients were exponentiated to derive adjusted fold-change estimates with 95% confidence intervals (CI). Classification performance was assessed using logistic regression with stratified 5-fold cross-validation. In each fold, models were trained on 80% of the data and evaluated on the remaining 20%, preserving class proportions. Continuous predictors were standardized within each training fold and applied to the corresponding test fold. Receiver operating characteristic (ROC) curves were generated from predicted probabilities, and mean area under the curve (AUC) ± standard deviation across folds was reported. Analyses were conducted using complete-case data without imputation.

## Results

### Study cohort and clinical characterization

The study included 206 participants comprising 101 patients with cognitive impairment and 105 cognitively healthy controls (HC) (Table 1). Patients were older than HC (70.6 ± 8.8 vs. 52.9 ± 18.2 years, *p* < 0.0001), whereas sex distribution was comparable between groups. Years of education were numerically lower in patients than in HC, but this difference did not reach statistical significance after recalculation using the updated individual-level dataset (14.8 ± 3.8 vs. 15.8 ± 3.2 years, *p* = 0.054). As expected, patients showed significantly impaired cognitive performance (DemTect) and increased functional impairment (FAQ), accompanied by higher subjective cognitive complaints (MAC-Q) and depressive symptoms (BDI-II) (all *p* ≤ 0.0005; Table 1). Constipation severity (CCCSS) did not differ between groups. Within the patient cohort, 16 individuals (15.8%) were diagnosed with dementia, 82 (81.2%) with mild cognitive impairment (MCI), and three (3.0%) with subjective cognitive decline (SCD). Across syndromes, 65 patients were classified as having Alzheimer’s disease (AD). Diagnoses were established using standardized neuropsychological assessment and biomarker-supported criteria, including CSF measurements (Aβ_1–42_, Aβ_1–40_, total tau, phosphorylated tau181) and, where available, amyloid and tau PET imaging (Additional file 2: Table S1). Given the age difference between groups, subsequent analyses included age-adjusted models and analyses using an age-restricted control subset. Patients were further stratified according to CSF- and/or PET-derived AT status into A(−)T(−), A(+)T(−), A(−)T(+), and A(+)T(+) groups to enable evaluation of fecal aggregate concentrations relative to canonical amyloid and tau biomarker classification.

**Table 1:** Demographic and clinical characteristics of the study cohort

Variable	HC (*n* = 105)	Patients (*n* = 101)	*p* value
Female, n (%)	63 (60.0%)	56 (55.4%)	0.603
Age, years (mean ± SD)	52.9 ± 18.2	70.6 ± 8.8	< 0.0001
Education, years (mean ± SD)	15.8 ± 3.2	14.8 ± 3.8	0.054
CCCSS (mean ± SD)	2.5 ± 3.3	2.2 ± 2.9	0.497
DemTect (mean ± SD)	16.4 ± 2.2	11.5 ± 4.1	< 0.0001
BDI-II (mean ± SD)	6.7 ± 7.4	11.0 ± 9.5	0.0005
MAC-Q (mean ± SD)	22.5 ± 4.2	28.3 ± 4.0	< 0.0001
FAQ (mean ± SD)	0.7 ± 2.4	6.2 ± 7.1	< 0.0001

*CCCSS* Cleveland Clinic Constipation Scoring System; *BDI-II* Beck Depression Inventory II, *MAC-Q* Memory Assessment Clinic-Questionnaire, *FAQ* Functional Activities Questionnaire, *NA* Not available

*P *values were calculated using the chi-square test for categorical variables and Welch’s t-test for continuous variables

### Fecal protein aggregates are elevated in cognitive impairment and remain elevated after age restriction

**Fig. 1:**
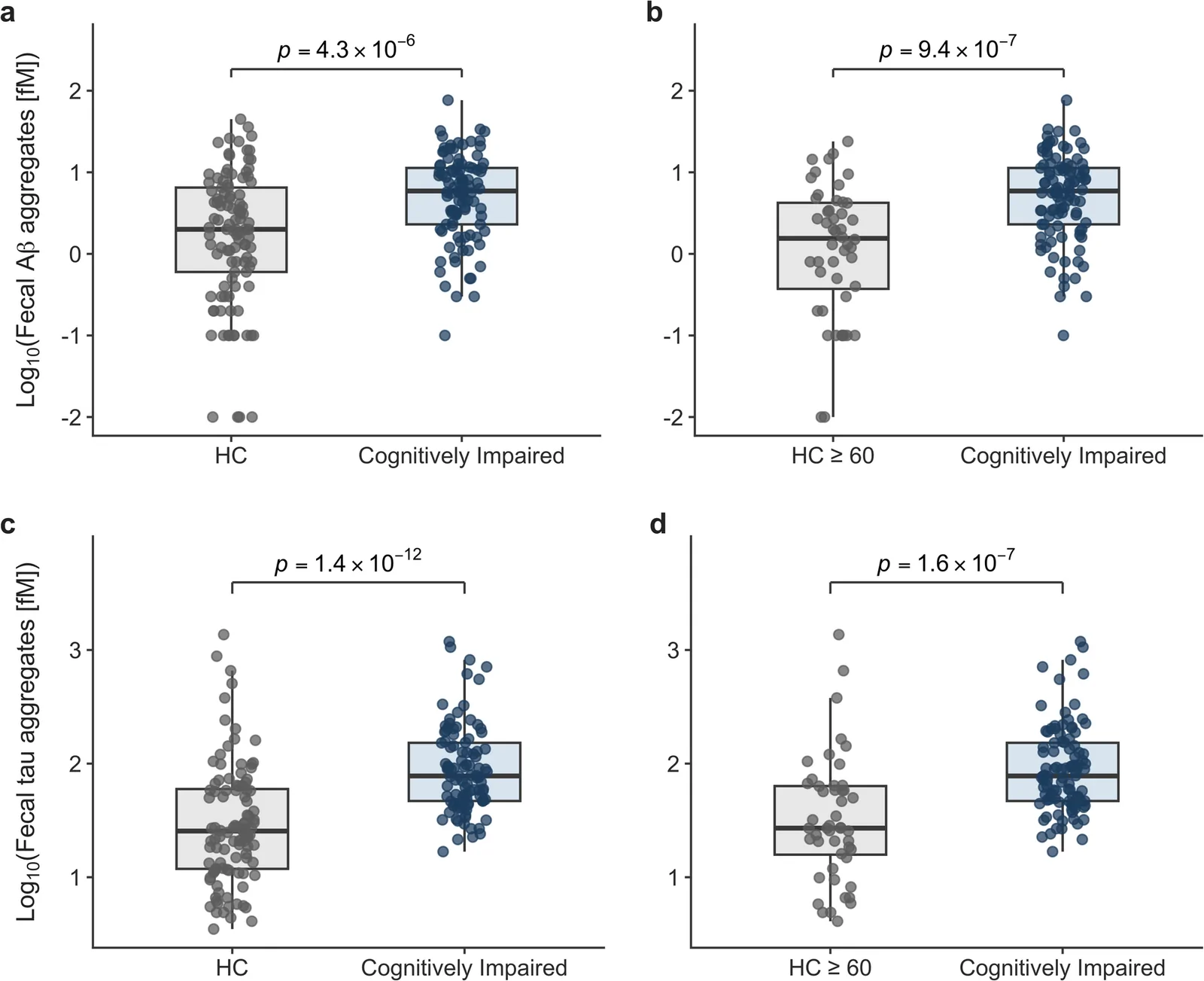
Fecal Aβ and tau aggregate burdens are elevated in cognitive impairment and remain elevated after age restriction. **a**, **c** Log_10_-transformed fecal Aβ (**a**) and tau (**c**) aggregate concentrations in cognitively healthy controls (HC) and patients with cognitive impairment. **b**, **d** Corresponding age-restricted analyses using controls aged ≥ 60 years for Aβ (**b**) and tau (**d**). Fecal Aβ and tau aggregates are increased in patients compared with controls in the full cohort and remain significantly elevated after restricting controls to individuals aged ≥ 60 years, supporting an association that is not explained by age imbalance alone. Data are shown as box plots (median and interquartile range; whiskers indicate Tukey limits) with individual observations. Statistical comparisons were performed using two-sided Mann-Whitney U tests; exact *p*-values are shown in the figure

Fecal Aβ and tau aggregate concentrations were significantly elevated in patients with cognitive impairment (dementia/MCI/SCD) compared with cognitively healthy controls (HC). Patients (*n* = 101) exhibited a median fecal Aβ concentration of 5.90 fM (IQR 2.30–11.30) vs. 2.00 fM (0.60–6.50) in HC (*n* = 105; Mann-Whitney U *p* = 4.3 × 10⁻^6^; Cliff’s δ = 0.37, moderate effect size) (Fig. 1a). For fecal tau aggregates, patients (*n* = 100) showed a median concentration of 77.85 fM (46.90–152.43) compared with 25.50 fM (11.85–59.77) in HC (*n* = 104; *p* = 1.4 × 10⁻^12^; Cliff’s δ = 0.58, large effect size) (Fig. 1c). To address potential age confounding, the primary patient-control analysis was additionally presented using an age-restricted control subset aged ≥ 60 years (*n* = 48; Fig. 1b and d). This subset had a mean age of 68.9 ± 6.4 years, a median age of 69.0 years (IQR 63.8–73.0; range 60–83), included 31 women and 17 men, and had a mean education of 15.8 ± 3.6 years among participants with available education data (*n* = 46). Compared with the patient group, the age-restricted HC subset no longer differed significantly in age (68.9 ± 6.4 vs. 70.6 ± 8.8 years, *p* = 0.187). Group differences remained significant for both fecal Aβ (*p* = 9.4 × 10⁻^7^; Cliff’s δ = 0.50, large effect size) and fecal tau (*p* = 1.6 × 10⁻^7^; Cliff’s δ = 0.53, large effect size). The persistence of these effects in the age-restricted analysis indicates that the observed differences are not explained by age imbalance alone. Global Kruskal–Wallis analyses across HC, SCD, MCI, and dementia groups were significant for both fecal Aβ and fecal tau, including after restricting controls to HC ≥ 60 years (Additional file 3: Fig. S2). Exploratory pairwise Mann–Whitney U tests showed no significant differences among the patient clinical subgroups, namely SCD, MCI, and dementia, for either analyte. Compared with HC, MCI and dementia showed significantly higher fecal Aβ and tau aggregate concentrations. SCD did not differ significantly from HC for fecal Aβ, and a nominal difference for fecal tau in SCD versus the full HC group did not persist in the age-restricted HC comparison. Given the small SCD subgroup (*n* = 3), these pairwise comparisons should be interpreted cautiously.

### Fecal Aβ and tau aggregates are elevated in cognitive impairment independently of age and sex

In multivariable linear regression models of log_10_-transformed fecal concentrations adjusted for age and sex, cognitive impairment remained strongly associated with increased fecal aggregate burden (Additional file 4: Fig. S3). Compared with cognitively healthy controls, patients exhibited a 4.18-fold increase in fecal Aβ concentrations (95% CI 2.52–6.95, *p* = 1.05 × 10⁻^7^) and a 2.97-fold increase in fecal tau concentrations (95% CI 2.11–4.20, *p* = 3.41 × 10⁻^9^). These findings demonstrate robust, demographic-independent elevations of fecal protein aggregates in cognitive impairment. Additional sensitivity analyses including years of education as a covariate yielded similar results. Cognitive impairment remained associated with higher fecal Aβ concentrations (4.15-fold increase, 95% CI 2.42–7.13, *p* = 5.5 × 10⁻^7^) and higher fecal tau concentrations (2.88-fold increase, 95% CI 1.99–4.15, *p* = 5.3 × 10⁻^8^), indicating that the observed fecal aggregate elevations were not explained by differences in educational attainment.

### Fecal aggregate elevations are not restricted to CSF-defined AT-positive patients

**Fig. 2:**
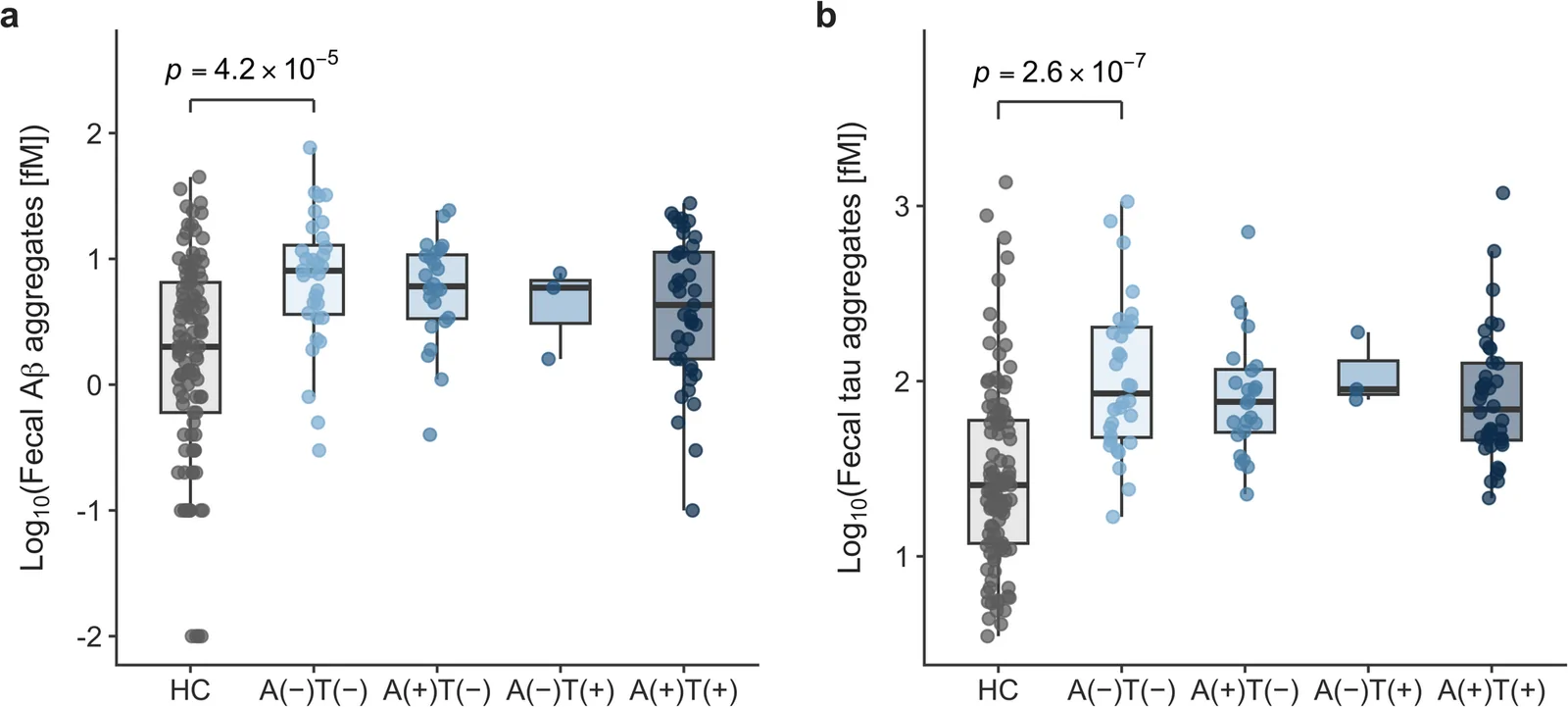
Fecal Aβ and tau aggregate elevations are not restricted to CSF biomarker-positive individuals and extend across AT-defined strata. **a**, **b** Log_10_-transformed fecal Aβ (**a**) and tau (**b**) aggregate concentrations across cognitively healthy controls (HC) and CSF-defined AT biomarker categories. Elevated fecal aggregate levels are observed even in biomarker-negative patients (A(−)T(−)), and group differences across AT strata indicate that fecal aggregate burden is not confined to canonical amyloid- or tau-positive disease states. Data are shown as box plots (median and interquartile range; whiskers indicate Tukey limits) with individual observations. Annotated *p*-values represent the pairwise comparisons between HC and the A(−)T(−) subgroup using two-sided Mann–Whitney U tests; other pairwise comparisons are not annotated

Importantly, fecal aggregate elevations were observed even in biomarker-negative patients (A(−)T(−), *n* = 32). Mean ages across the AT-defined patient subgroups were 66.8 ± 9.7 years for A(−)T(−), 71.3 ± 7.6 years for A(+)T(−), 71.7 ± 12.7 years for A(−)T(+), and 72.9 ± 7.9 years for A(+)T(+); the mean age of HC was 52.9 ± 18.2 years. Age was unavailable for one patient in the A(+)T(−) subgroup. In the A(−)T(−) subgroup, fecal Aβ aggregates were 8.05 fM (3.62–12.88), significantly higher than 2.00 (0.60–6.50) fM in HC (*p* = 4.2 × 10⁻^5^; Cliff’s δ = 0.48) (Fig. 2a). Fecal tau aggregates were likewise elevated with 85.45 fM (47.73–203.22) vs. 25.50 (11.85–59.77) fM in HC (*p* = 2.6 × 10⁻⁷; Cliff’s δ = 0.60) (Fig. 2b). Across AT strata, global differences were significant for both fecal Aβ and fecal tau (Kruskal-Wallis *p* < 0.001). These findings were consistent when restricting the analysis to controls aged ≥ 60 years (Additional file 5: Fig. S4). These findings indicate that fecal aggregate elevations are not limited to patients with CSF/PET-defined amyloid or tau positivity. Because AT status was not available for HC, this analysis should be interpreted as exploratory and as a comparison between biomarker-characterized patient subgroups and cognitively healthy controls, rather than confirmed biomarker-negative controls.

### Fecal Aβ, but not fecal tau, inversely associates with CSF tau pathology markers in patients

**Fig. 3:**
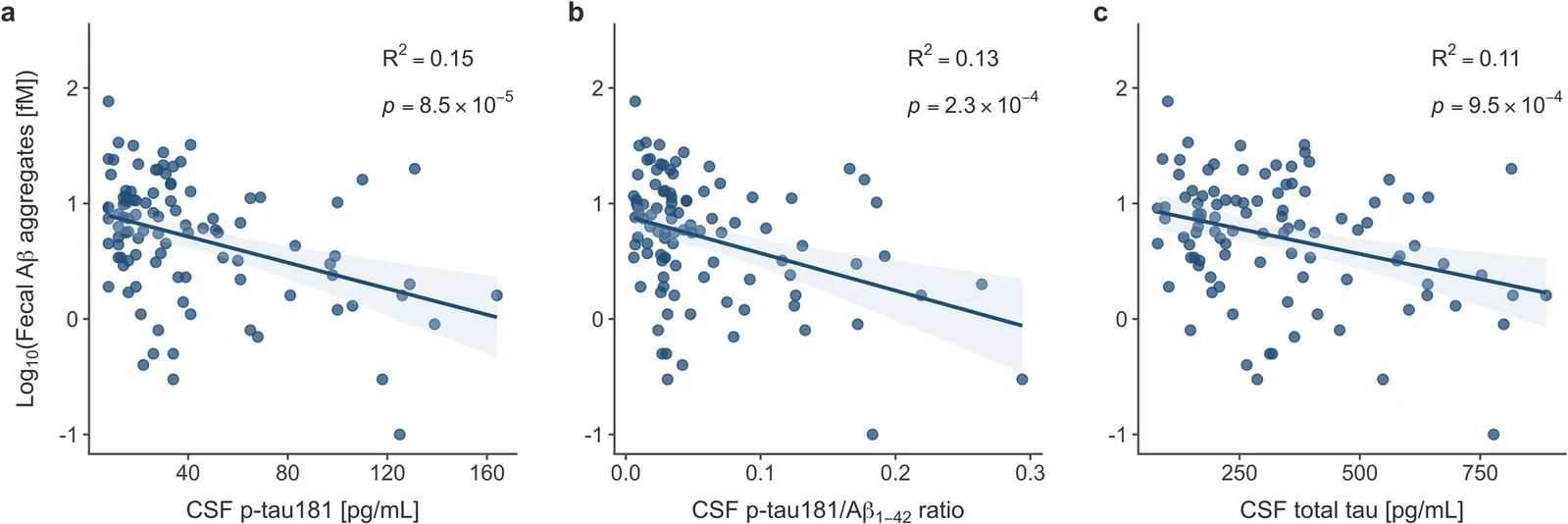
Fecal Aβ aggregate levels inversely associate with CSF tau biomarkers. **a**–**c** Log_10_-transformed fecal Aβ aggregate concentrations plotted against CSF phosphorylated tau181 (p-tau181) (**a**), the CSF p-tau181/Aβ1–42 ratio (**b**), and CSF total tau (**c**). Across all measures, fecal Aβ aggregates show consistent inverse associations with central tau-related biomarkers. Each dot represents one participant (*n* = 99). Solid lines indicate linear regression fits; shaded areas represent 95% confidence intervals. Coefficients of determination (R^2^) and two-sided *p*-values from linear regression are shown in the panels

Within the patient cohort, log_10_-transformed fecal Aβ aggregate concentrations showed inverse associations with CSF tau markers. Significant negative associations were observed with CSF p-tau181 (R^2^ = 0.15, *p* < 0.0001, *n* = 99) in univariable linear regression analyses (Fig. 3a) and the p-tau181/Aβ_1–42_ ratio (R^2^ = 0.13, *p* = 0.0002, *n* = 99) (Fig. 3b). A weaker but still significant inverse association was detected with CSF total tau (R^2^ = 0.11, *p* < 0.001, *n* = 99) (Fig. 3c). In multivariable linear regression models adjusting for age, sex, and clinical AD diagnosis (AD vs. non-AD), CSF p-tau181 remained an independent negative predictor of log_10_-transformed fecal Aβ concentrations (β = −0.0054 per pg/mL, *p* < 0.001, *n* = 99). These findings indicate that fecal Aβ aggregate burden is inversely associated with CSF tau biomarkers, particularly phosphorylated tau, independent of demographic factors and clinical AD status.

By contrast, log_10_-transformed fecal tau aggregate concentrations showed no significant associations with CSF total tau, CSF p-tau181, or the CSF p-tau181/Aβ_1–42_ ratio. Thus, the inverse association with CSF tau-related biomarkers was observed for fecal Aβ aggregates, but not for fecal tau aggregates.

### Independent associations of fecal aggregates with cognitive impairment in logistic regression models

**Fig. 4:**
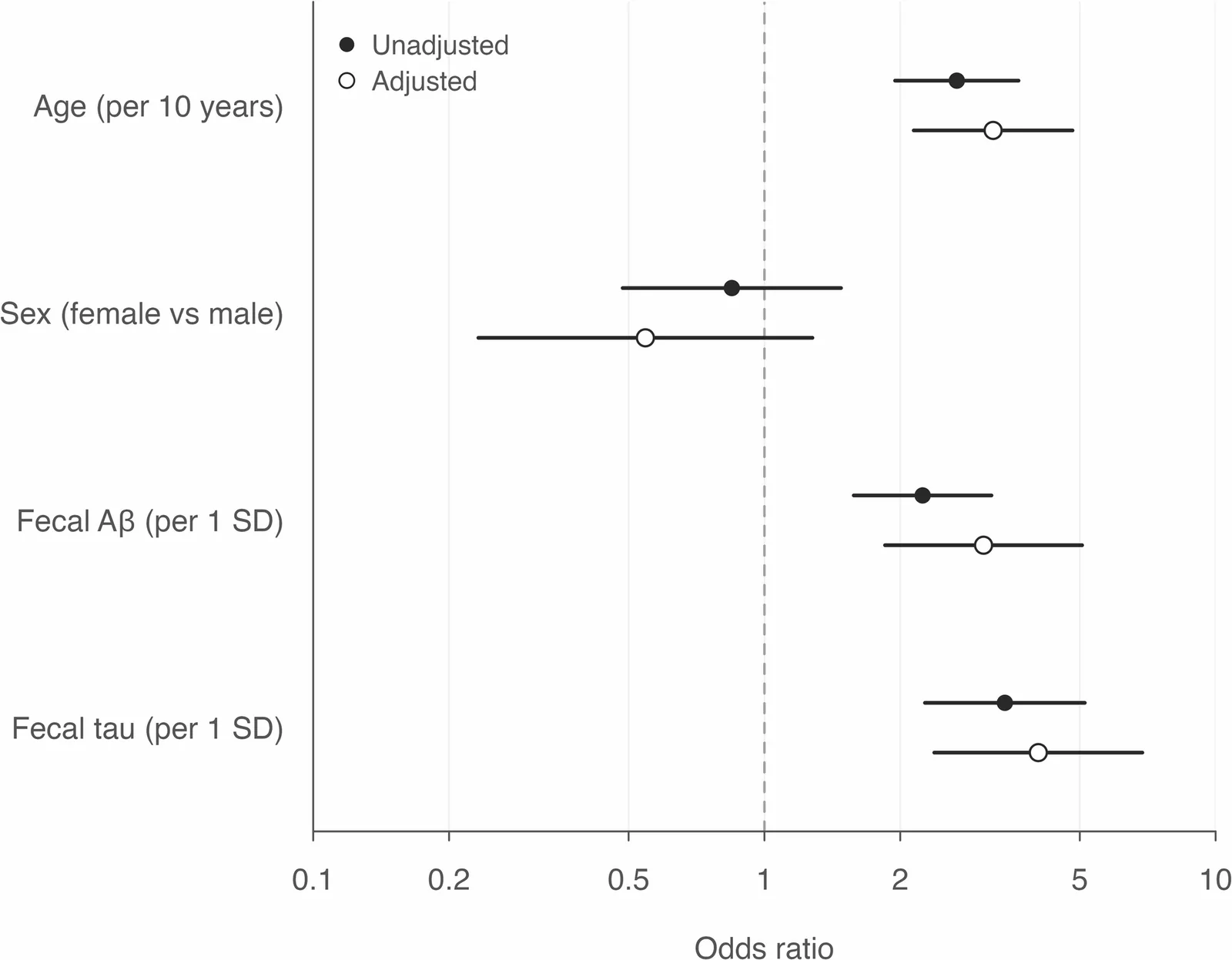
Fecal tau and Aβ aggregates independently associate with cognitive impairment beyond demographic factors. Forest plot showing odds ratios (ORs) and 95% confidence intervals (CIs) for the association between cognitive impairment (patients vs. cognitively healthy controls) and demographic and fecal aggregate predictors. Unadjusted estimates (●) are derived from single-predictor logistic regression models. Adjusted estimates (○) are derived from the multivariable model including age (per 10 years), sex (female vs. male), log_10_-transformed fecal tau (per 1 SD), and log_10_-transformed fecal Aβ (per 1 SD). Both fecal biomarkers remain associated with cognitive impairment after adjustment, indicating that fecal aggregate burden provides information beyond established demographic risk factors. The vertical reference line indicates an OR of 1 (no association). Odds ratios are displayed on a logarithmic scale. Multivariable estimates were derived from complete-case analyses (*n* = 203)

Logistic regression analyses were performed in the study cohort (*n* = 206) to assess associations between demographic variables, fecal aggregate measures, and cognitive impairment (Fig. 4). In univariable logistic regression models, increasing age was significantly associated with patient status (β = 0.098 per year, OR = 1.10 per year; equivalent to OR = 2.66 per 10 years; *p* = 1.0 × 10⁻^9^). Higher log_10_-transformed fecal tau aggregate concentrations (β = 2.35, OR = 10.5 per 10-fold increase; *p* = 3.7 × 10⁻^9^) and fecal Aβ aggregate concentrations (β = 1.10, OR = 3.00 per 10-fold increase; *p* = 6.7 × 10⁻^6^) were also significantly associated with patient status. Because logistic regression coefficients depend on predictor scaling, the absolute β values should not be directly compared across variables measured in different units. Sex was not associated with cognitive impairment.

In multivariable logistic regression models including age, sex, log_10_-transformed fecal tau, and log_10_-transformed fecal Aβ concentrations, both fecal tau (β = 2.68, OR = 14.6, 95% CI 5.64–43.85, *p* = 2.5 × 10⁻⁷) and fecal Aβ (β = 1.53, OR = 4.61, 95% CI 2.44–9.74, *p* = 1.3 × 10⁻⁵) remained independently associated with cognitive impairment, with fecal tau showing the strongest effect. Age also remained a significant independent predictor, whereas sex did not. Multivariable analyses were based on complete cases (*n* = 203). Sensitivity analyses additionally including years of education as a covariate yielded similar results. Fecal tau (OR = 11.60, 95% CI 4.20–32.06, *p* = 2.3 × 10⁻^6^) and fecal Aβ (OR = 4.26, 95% CI 2.13–8.52, *p* = 4.2 × 10⁻^5^) remained independently associated with cognitive impairment. Education itself was not associated with patient status in this model (OR = 0.98 per year, 95% CI 0.86–1.12, *p* = 0.773; complete cases, *n* = 189).

**Fig. 5:**
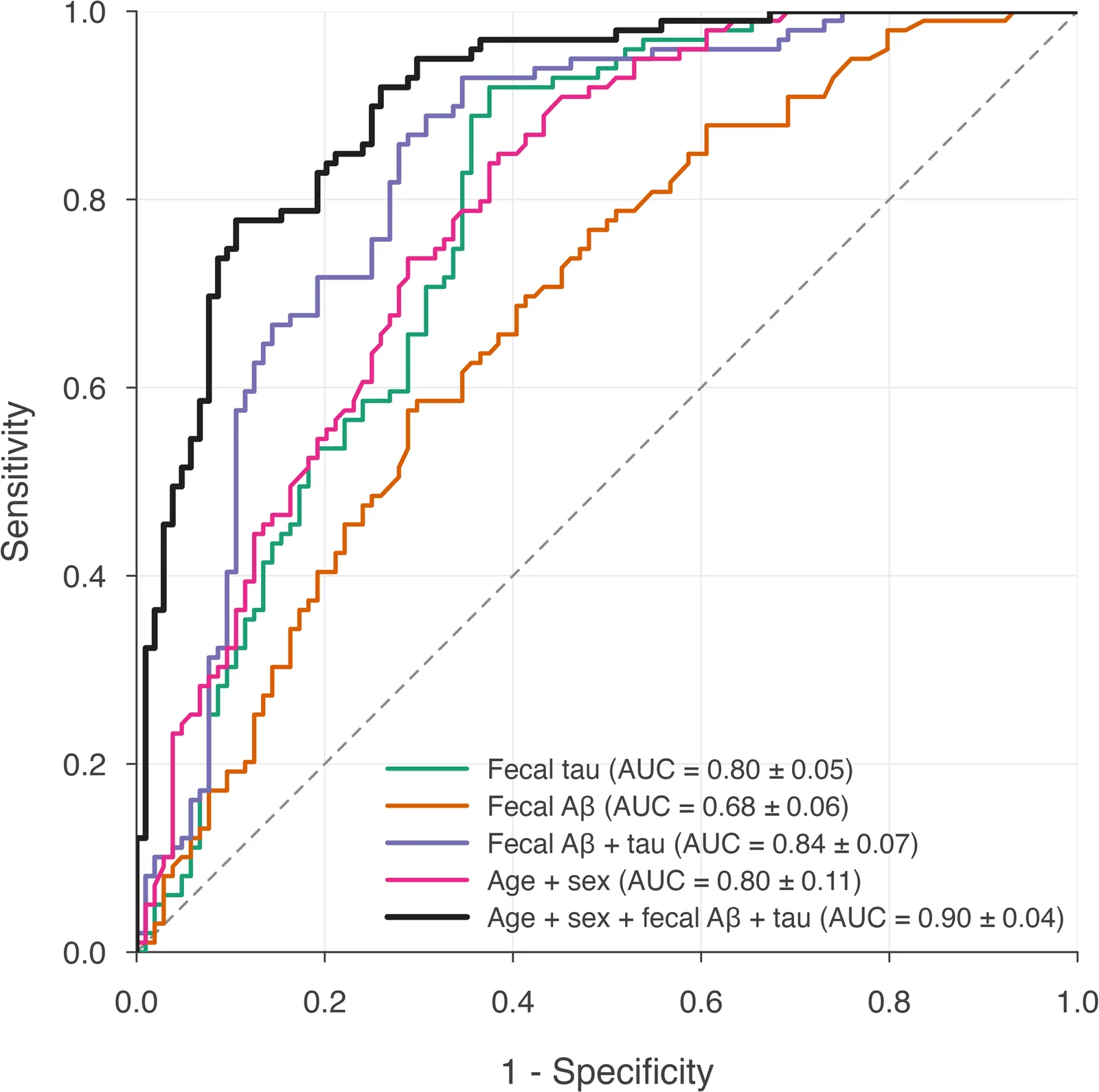
Fecal aggregate biomarkers improve discrimination of cognitive impairment beyond demographic variables. Receiver operating characteristic (ROC) curves for classification of cognitively impaired patients versus cognitively healthy controls using logistic regression models based on fecal tau, fecal Aβ, their combination, demographic variables (age and sex), and a combined model including both demographic and fecal predictors. Fecal tau showed stronger discriminative performance than fecal Aβ (AUC ~ 0.80 vs. ~ 0.68), and combining both markers improved classification (AUC ~ 0.84). Demographic variables alone yielded moderate discrimination (AUC ~ 0.80), whereas integration of fecal biomarkers further increased performance (AUC ~ 0.90), indicating substantial incremental diagnostic value beyond age and sex. Curves are derived from stratified 5-fold cross-validated models. The diagonal line indicates no discriminative performance (AUC = 0.5)

To evaluate classification performance, logistic regression models were fitted using participants with complete data for the variables included in each model. Stratified 5-fold cross-validation was applied to preserve the proportion of patients and controls across folds. In each iteration, the model was trained on four folds, corresponding to approximately 80% of the complete-case dataset, and tested on the remaining fold. This procedure was repeated until each fold had served once as the test set, and classification performance was summarized using receiver operating characteristic curves and the area under the curve. Among models using fecal biomarkers alone, log_10_-transformed fecal tau aggregates showed robust discriminative performance (AUC = 0.80 ± 0.05, 95% CI 0.72–0.85), outperforming fecal Aβ (AUC = 0.68 ± 0.06, 95% CI 0.61–0.75) (Fig. 5). Combining fecal Aβ and tau improved classification (AUC = 0.84 ± 0.07, 95% CI 0.77–0.89). Demographic variables alone (age and sex) achieved similar mean performance (AUC = 0.80 ± 0.11, 95% CI 0.73–0.85) but exhibited greater variability across folds. Integration of fecal aggregates with demographic predictors yielded the highest and most consistent discrimination (AUC = 0.90 ± 0.04, 95% CI 0.87–0.95), indicating substantial incremental diagnostic value beyond demographic variables. Because age and sex alone showed substantial classification performance in the full cohort, we repeated the cross-validated diagnostic analyses using all patients and restricting the control group to individuals aged ≥ 60 years. In this more age-balanced population, demographic variables alone no longer showed strong discrimination (AUC = 0.58 ± 0.12). In contrast, fecal aggregate biomarkers retained discriminatory performance, with AUCs of 0.77 ± 0.08 for fecal tau, 0.75 ± 0.08 for fecal Aβ, and 0.84 ± 0.03 for the combined fecal Aβ/tau model. Adding age and sex to the fecal biomarker model yielded similar performance (AUC = 0.84 ± 0.05; complete cases, *n* = 147), indicating that the biomarker signal was not primarily driven by the age and sex structure of the full cohort.

## Discussion

AD and related cognitive disorders are increasingly considered in the context of dynamic interactions between central proteopathic processes and peripheral biological pathways [5, 13, 14]. In this study, we demonstrate that aggregates of both Aβ and tau are detectable in human fecal samples and that their concentrations are significantly elevated in individuals with cognitive impairment compared with cognitively healthy controls. These findings extend the emerging concept that pathological protein aggregation associated with AD is not confined to the CNS but can also be detected in peripheral biological compartments. Importantly, these differences remained robust after adjustment for demographic variables and after restricting controls to individuals aged ≥ 60 years, indicating that the observed signal cannot be explained by age differences alone. The detection of fecal tau aggregates represents a substantial extension of previous work on stool-based Aβ detection and suggests that multiple classes of disease-associated aggregates can be quantified in fecal material [22, 23, 26, 40].

Our earlier proof-of-concept study reported elevated fecal Aβ aggregates in AD using sFIDA [22]. The present data extend this concept in three important ways. First, we show that tau aggregates are detectable in feces and elevated in cognitive impairment, broadening the biological scope from amyloid alone to the two core aggregate classes that define AD pathology. Second, combining fecal Aβ and fecal tau improves discrimination relative to single markers, consistent with the general principle that multi-analyte aggregate profiling often outperforms individual biomarkers. Third, fecal aggregate elevations were observed even in individuals without CSF-defined AT biomarker positivity, indicating that stool-based aggregate measurements may capture aspects of systemic proteopathic biology that are not fully reflected by canonical central biomarkers. These findings are conceptually aligned with previous applications of sFIDA in biofluids, where quantitative aggregate measurements have proven informative across several neurodegenerative proteinopathies [28, 29, 40–42]. The scope of these findings should be interpreted in light of the study population. Although Aβ and tau are central proteins in AD pathobiology and 65 patients in the cohort were classified as having AD, the primary analyses were performed in a clinically heterogeneous group of cognitively impaired individuals that also included non-AD diagnoses. Therefore, the present data should not be interpreted as demonstrating an AD-specific stool biomarker signature. Rather, they indicate that fecal Aβ and tau aggregate concentrations are elevated in cognitive impairment and may reflect a broader peripheral proteopathic process that overlaps with, but is not restricted to, biomarker-defined AD. Larger cohorts specifically powered for AT-defined and etiologically defined subgroup analyses will be required to determine the disease specificity of fecal aggregate signatures.

Our findings support the concept that protein aggregation associated with neurodegenerative disease is not confined to the CNS but may also be reflected in peripheral compartments [26]. Increasing evidence indicates that clearance of Aβ involves a network of processes that include glymphatic drainage, blood-brain barrier transport, and peripheral elimination mechanisms [13]. In particular, hepatobiliary pathways have been implicated in the systemic clearance of circulating Aβ, with the liver acting as a major site of uptake and degradation before secretion into bile and subsequent intestinal elimination [16]. These mechanisms provide a biologically plausible framework through which aggregated proteins may enter peripheral elimination pathways and ultimately become detectable in fecal material. Although the precise origin of fecal aggregates cannot be determined from the present data, both hepatobiliary clearance of circulating aggregates and local processes within the enteric nervous system may contribute to their appearance in stool [23, 24].

The rationale for analyzing stool is therefore distinct from, and complementary to, blood-based biomarker approaches. Plasma Aβ and phosphorylated tau biomarkers provide highly valuable information about brain amyloid and tau pathology and are increasingly used as scalable screening tools. In contrast, fecal aggregate measurements may capture proteins that have entered gastrointestinal or hepatobiliary elimination pathways, or aggregates associated with local intestinal and enteric nervous system biology. Stool may therefore provide information about peripheral aggregate handling, clearance, and gut–brain-axis biology that is not directly accessible from plasma measurements alone. Thus, fecal aggregate quantification should not be viewed as a replacement for established blood-based biomarkers, but as a complementary approach that may help characterize systemic and gastrointestinal dimensions of proteopathic disease.

A notable observation in this study is that fecal aggregate elevations were not restricted to individuals with CSF-defined AT positivity. Elevated fecal Aβ and tau concentrations were also detected in the A(−)T(−) subgroup, indicating that stool aggregate burden is not simply a downstream reflection of canonical amyloid and tau biomarkers measured in CSF. One interpretation is that fecal aggregates capture systemic proteopathic dynamics that extend beyond the central AT cascade. Peripheral handling of aggregated proteins may vary between individuals depending on metabolic, inflammatory, and gastrointestinal factors. Importantly, clinical cohorts of cognitively impaired individuals frequently include heterogeneous or mixed underlying pathologies, and AT-negative patients may represent early disease stages or non-AD proteinopathies that are not captured by CSF Aβ and tau biomarkers alone [2, 4, 8, 9, 43, 44]. The persistence of fecal aggregate elevations in these subgroups therefore suggests that stool-based aggregate measurements may represent a complementary biological dimension rather than a direct surrogate for CNS biomarker status.

A limitation of this analysis is that AT biomarker status was not available for the cognitively healthy control group. Therefore, some HC individuals may have had preclinical amyloid or tau pathology despite normal cognition. This limitation may attenuate differences between HC and AT-defined patient subgroups and precludes interpretation of fecal aggregates as fully independent of central AT pathology. The significant elevation of fecal Aβ and tau aggregates in A(−)T(−) patients should therefore be interpreted cautiously. Rather than indicating AD pathology in AT-negative individuals, this finding suggests that stool-based aggregate measurements may capture biological information not fully represented by CSF/PET-defined amyloid and tau biomarkers, including systemic proteopathic processes, mixed or non-AD pathology, or peripheral aggregate handling and clearance.

The inverse association observed between fecal Aβ aggregates and CSF tau measures represents an additional indication that fecal aggregates reflect biological processes distinct from those captured by central biomarkers. CSF phosphorylated tau and total tau are widely interpreted as indicators of neuronal injury and tau pathology and increase with disease progression along the AD continuum [2, 3, 8, 9, 45, 46]. Although the strength of these associations is moderate (R^2^ ≈ 0.11–0.15), the consistency across multiple tau-related biomarkers suggests that fecal aggregate signals capture biological information that is not redundant with established CSF markers. Because CSF tau biomarkers are reported in pg/mL, the regression coefficient per 1 pg/mL is numerically small; however, across clinically relevant differences in CSF p-tau181 or total tau concentrations, the corresponding change in fecal Aβ aggregate burden becomes more appreciable. For example, the β estimate for CSF p-tau181 (− 0.0054 per pg/mL) corresponds to an approximately 0.054 log_10_-unit lower fecal Aβ concentration per 10 pg/mL higher CSF p-tau181, equivalent to approximately 12% lower fecal Aβ, and to an approximately 0.27 log_10_-unit lower value across a 50 pg/mL difference, equivalent to approximately 46% lower fecal Aβ. This association should not be interpreted as a direct stoichiometric coupling between CSF tau and fecal Aβ. Rather, together with the absence of uniformly increased fecal Aβ concentrations across all T-positive subgroups, the modest R^2^ values support the interpretation that fecal Aβ aggregates are not a direct surrogate of central tau pathology but reflect partly distinct peripheral or systemic processes. Such processes may include systemic elimination or redistribution pathways operating outside the CNS. One possible explanation is that fecal aggregates reflect peripheral handling or elimination processes that may vary across disease stages. Under such a model, increasing central tau pathology could coincide with altered peripheral distribution or clearance of aggregated species, resulting in the inverse association observed between fecal Aβ aggregates and CSF tau measures. Alternatively, fecal aggregate concentrations may be influenced by gastrointestinal physiology, including intestinal transit time, bile secretion, microbial protease activity, and stool matrix composition. Such factors could modulate the stability or detectability of aggregated proteins within the intestinal lumen and may therefore contribute to fecal aggregate variability independent of central pathology. Although constipation severity measured by the CCCSS did not differ between groups in this cohort (Table 1), this metric provides only a coarse assessment of gastrointestinal function and does not capture additional biological factors such as bile acid metabolism or microbiome composition that may influence intestinal aggregate levels [47–49].

These findings intersect with growing interest in the gut-brain axis in neurodegenerative disease [17, 47]. Bidirectional communication between the gastrointestinal tract and the CNS occurs through neural, immune, endocrine, and microbial pathways, and alterations in gut microbiota composition, intestinal barrier function, and systemic inflammation have been reported in AD and prodromal disease stages [18–20]. Although microbiome composition was not examined in the present study, the detection of disease-associated protein aggregates in stool adds a complementary molecular dimension to this emerging field. Stool aggregate measurements may therefore provide a means of linking gastrointestinal biology with systemic proteostasis and neurodegenerative pathology.

From a translational perspective, the diagnostic analyses highlight both the potential and current limitations of stool-based aggregate biomarkers. Fecal tau aggregates showed stronger discriminatory performance than fecal Aβ alone, and combining both markers further improved classification accuracy. The strongest performance was observed when fecal aggregates were integrated with demographic predictors, suggesting that stool aggregate measurements provide incremental diagnostic information within multimodal biomarker frameworks [2]. In clinical settings, such markers may be most useful as minimally invasive screening tools that help identify individuals who would benefit from confirmatory CSF, imaging-based, or blood-based diagnostics [8, 45, 46, 50]. However, the observed overlap between groups indicates that stool-based aggregate measurements are unlikely to replace established biomarkers. Instead, they may complement existing CSF, imaging, and plasma biomarker approaches by providing information about peripheral aggregate handling and gastrointestinal or hepatobiliary pathways.

The diagnostic analyses should also be interpreted in light of the age imbalance in the full cohort. Although the full-cohort model including age and sex showed high classification performance, this was largely attributable to the marked age difference between patients and controls. When diagnostic models were repeated in the age-restricted dataset, the performance of age and sex alone decreased substantially, whereas fecal Aβ and tau aggregate measurements retained discriminatory value. Nevertheless, these analyses do not replace validation in a prospectively age-matched cohort, and future studies should evaluate diagnostic performance in independent cohorts with balanced demographic characteristics.

Several aspects of the study strengthen the interpretation of these findings. The sFIDA platform allows highly selective detection of aggregated protein species while minimizing interference from monomeric peptides, enabling quantification of aggregates at sub-femtomolar concentrations [27]. The integration of stool-based measurements with CSF biomarker data and AT classification also provides a direct comparison with established biological definitions of AD. Furthermore, the persistence of group differences after demographic adjustment and age-restricted analyses supports the robustness of the observed associations.

A further limitation is that the cognitively healthy controls were recruited as a broad reference cohort for fecal aggregate measurements rather than as a prospectively age-matched elderly control group. Consequently, the full HC group included substantially younger individuals than the patient cohort, which may raise concerns about age-related confounding. To mitigate this limitation, we presented age-restricted analyses using controls aged ≥ 60 years as part of the main patient-control comparison. In this restricted subset, the age distribution was substantially more comparable to that of the patient group, and fecal Aβ and tau aggregate concentrations remained significantly elevated in patients. Nevertheless, this analysis does not fully replace a prospectively age-matched control cohort, and future validation studies should include elderly cognitively healthy controls selected to match the patient cohort more closely in age and other demographic characteristics.

At the same time, several considerations should be acknowledged. Stool biomarkers are inherently influenced by pre-analytical variables, including diet, medications, microbial composition, and sample handling, and standardized protocols will be essential for future multicenter validation studies [51–54]. In addition, the clinical cohort included heterogeneous cognitive diagnoses, which may contribute biological variability but also reflects the complexity of real-world patient populations. Finally, the mechanistic relationship between central aggregate pathology and fecal aggregate concentrations remains to be fully elucidated and will require further investigation.

Although diagnostic performance was assessed using stratified 5-fold cross-validation, this approach provides internal validation only and does not replace testing in an independent external cohort. Therefore, the classification results should be interpreted as exploratory estimates of diagnostic performance. Future studies should validate fecal Aβ and tau aggregate measurements in independent, preferably multicenter cohorts to determine their generalizability, reproducibility, and clinical applicability, and should assess whether fecal aggregates change longitudinally across the AD continuum or in response to disease-modifying therapies. Integrating stool aggregate measurements with systemic markers—including liver function parameters, bile acid profiles, and microbiome composition—may further clarify the biological pathways linking peripheral aggregate handling with central neurodegenerative processes.

## Conclusions

This study demonstrates that both Aβ and tau aggregates can be quantified in human fecal samples and that their concentrations are significantly elevated in individuals with cognitive impairment. The detection of fecal tau aggregates extends previous observations on fecal Aβ and indicates that multiple classes of disease-associated protein aggregates can be measured in stool using single-particle assays [22]. The persistence of elevated fecal aggregates after demographic adjustment and in age-restricted analyses, together with their presence in AT biomarker-negative patients, suggests that fecal aggregate burden reflects systemic aspects of proteopathic biology that are not fully captured by CSF-defined amyloid and tau biomarkers. The observed inverse associations between fecal Aβ aggregates and CSF tau measures further support the notion that intestinal aggregate signals may relate to peripheral clearance or redistribution processes operating alongside central neurodegeneration. Collectively, these findings identify stool-based aggregate quantification as a promising minimally invasive approach for capturing systemic proteopathic alterations in cognitive impairment. With independent validation, longitudinal studies, and integration with established biomarkers, fecal aggregate measurements may contribute to scalable multimodal biomarker strategies for patient stratification and disease monitoring.

## Supplementary Information

Additional file 1: Figure S1 Representative sFIDA images of Aβ and tau aggregates

Additional file 2: Table S1 Individual-level demographic, clinical, CSF/PET biomarker, AT classification, and fecal aggregate data

Additional file 3: Figure S2 Fecal Aβ and tau aggregates remain elevated across clinical stages of cognitive impairment

Additional file 4: Figure S3 Demographic-adjusted associations between cognitive impairment and fecal aggregate concentrations

Additional file 5: Figure S4 Fecal Aβ and tau aggregate elevations across AT-defined strata persist after restriction to controls aged ≥60 years

## Data Availability

The images generated and/or analyzed during the current study are not publicly available but are available from the corresponding author on reasonable request. All other data generated or analyzed during this study are included in this published article [and its supplementary information files].

## Abbreviations

A: Amyloid deposition
AD: Alzheimer’s disease
ADL: Activities of daily living
Aβ: Amyloid-β
AUC: Area under the curve
BDI-II: Beck Depression Inventory II
BSA: Bovine serum albumin
CCCSS: Cleveland Clinic Constipation Scoring System
CI: Confidence interval
CSF: Cerebrospinal fluid
CV%: Coefficient of variation
Dem: Dementia
FAQ: Functional Activities Questionnaire
HC: Healthy controls
IQR: Interquartile range
MAC-Q: Memory Assessment Clinic Questionnaire
MCI: Mild cognitive impairment
N: Neurodegeneration
NIA-AA: National Institute on Aging-Alzheimer’s Association
PBS: Phosphate-buffered saline
PET: Positron emission tomography
ROC: Receiver operating characteristic
SCD: Subjective cognitive decline
SiNaPs: Silica nanoparticles
sFIDA: Surface-based fluorescence intensity distribution analysis
T: Tau pathology
TBS: Tris-buffered saline
TBST: Tris-buffered saline with 0.05% Tween 20
