# Supplementary material for: Fecal Aβ and tau aggregates are elevated in cognitive impairment and reveal peripheral proteopathic alterations
Source: Alzheimers Res Ther. 2026 Sep 26;18:215. doi: 10.1186/s13195-026-02188-0 (PMC13621666; doi:10.1186/s13195-026-02188-0)
Supplement: Supplementary file 1 [file 13195_2026_2188_MOESM1_ESM.pdf]

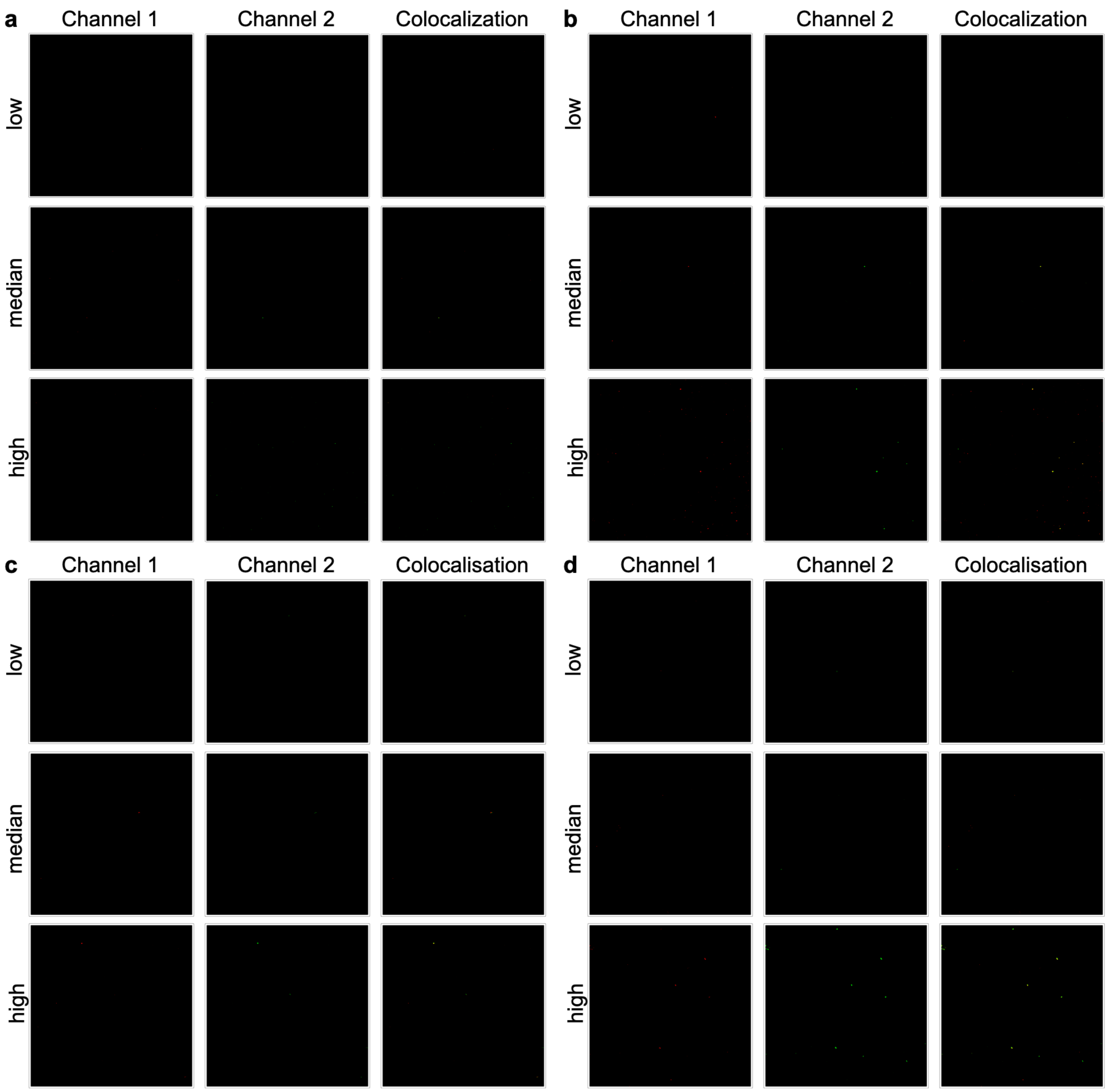

Figure S1 Representative sFIDA images of Aβ and tau aggregates. **a**–**d** Representative images of Aβ (**a, b**) and tau (**c, d**) aggregates in selected cognitively healthy control (HC; **a, c**) and patient (**b, d**) samples spanning low, median, and high sFIDA readout ranges. Samples were selected based on predefined quantitative readout positions rather than visual appearance. Images show, from left to right, fluorescence in Channel 1 (green), Channel 2 (red), and colocalized events, which were automatically quantified using sFIDAta. Because fecal aggregate concentrations

overlap between patients and controls, these images are intended to illustrate the assay signal and background characteristics, not to visually distinguish diagnostic groups. Quantitative analyses were based on automated analysis of all acquired images and technical replicates.
