# Supplementary material for: Fecal Aβ and tau aggregates are elevated in cognitive impairment and reveal peripheral proteopathic alterations
Source: Alzheimers Res Ther. 2026 Sep 26;18:215. doi: 10.1186/s13195-026-02188-0 (PMC13621666; doi:10.1186/s13195-026-02188-0)
Supplement: Supplementary file 2 [file 13195_2026_2188_MOESM2_ESM.pdf]

Table S1 Individual-level demographic, clinical, CSF/PET biomarker, AT classification, and fecal aggregate data.

Sample	Cohort	Clinical syndrome	Sex	Education (years)	Age at sampling (years)	CCCSS	DemTect	BDI-II	MAC-Q	FAQ	$A\beta_{1-42}$ (pg/mL)	$A\beta_{1-40}$ (pg/mL)	Ratio $A\beta_{1-42}$/ $A\beta_{1-40}$	Aβ amyloid status	Total tau (pg/mL)	p-tau181 (pg/mL)	Tau amyloid status	p-tau181/$A\beta_{1-42}$ ratio	Amyloid PET	Tau PET	Fecal Aβ aggregates (fM)	Fecal tau aggregates (fM)
1	Patient	MCI	F	NA	76	NA	NA	NA	NA	NA	851	9592	0.089	+	364	68	+	0.080	NA	NA	0.7	NA
2	Patient	MCI	M	NA	NA	NA	NA	NA	NA	NA	NA	NA	NA	+	NA	NA	-	NA	+	NA	12.0	89.2
3	Patient	Dem	F	NA	79	NA	NA	NA	NA	NA	515	6890	0.075	+	583	99	+	0.192	NA	NA	3.5	52.6
4	Patient	Dem	M	16	79	0	5	5	30	15	635	7483	0.085	+	614	83	+	0.131	NA	NA	4.3	32.0
5	Patient	MCI	M	12	57	1	4	10	28	3	1189	6285	0.189	-	185	27	-	0.023	NA	NA	19.6	94.0
6	Patient	MCI	F	15	70	1	4	6	24	0	621	10362	0.060	+	561	110	+	0.177	NA	NA	16.1	31.3
7	Patient	MCI	M	14	71	0	7	1	22	0	1138	12703	0.090	+	602	100	+	0.088	NA	NA	1.2	26.7
8	Patient	SCD	M	19	73	2	17	6	32	2	1513	7475	0.202	-	347	33	-	0.022	NA	NA	14.6	226.1
9	Patient	MCI	M	18	69	2	12	7	26	2	401	4961	0.081	+	548	118	+	0.294	NA	NA	0.3	156.1
10	Patient	MCI	F	11	75	5	10	9	31	5	806	8941	0.090	+	799	139	+	0.172	NA	NA	0.9	79.0
11	Patient	Dem	F	7	67	0	5	NA	34	26	787	8982	0.088	+	815	131	+	0.166	NA	NA	20.0	194.0
12	Patient	MCI	M	12	79	0	10	36	31	7	NA	NA	NA	NA	NA	NA	NA	NA	NA	NA	0.6	152.1
13	Patient	MCI	M	10	78	4	12	12	23	4	780	3375	0.231	-	330	26	-	0.033	NA	NA	12.3	75.6
14	Patient	MCI	F	12	81	0	16	6	24	4	664	6366	0.104	-	473	61	-	0.092	NA	NA	2.2	143.5
15	Patient	MCI	F	17	81	2	10	9	27	6	566	6935	0.082	+	674	97	+	0.171	NA	NA	3.0	46.9
16	Patient	MCI	M	20	65	1	9	9	33	2	488	6507	0.075	+	641	129	+	0.264	NA	NA	2.0	59.6
17	Patient	MCI	F	15	83	1	12	14	25	0	487	5272	0.092	+	458	65	+	0.133	NA	NA	0.8	66.2
18	Patient	MCI	F	9	69	3	15	8	23	0	888	5643	0.158	-	221	31	-	0.035	NA	NA	4.5	70.5
19	Patient	MCI	F	16	67	0	3	3	21	0	749	9276	0.081	+	887	164	+	0.219	NA	NA	1.6	43.3
20	Patient	MCI	F	14	73	0	11	6	24	4	806	12451	0.065	+	753	98	+	0.122	NA	NA	2.4	552.6
21	Patient	MCI	F	12	67	0	14	0	21	6	1171	7462	0.157	-	148	28	-	0.024	NA	NA	0.8	24.1

22	Patient	SCD	M	17	61	5	18	28	34	5	1094	6570	0.167	-	287	34	-	0.031	NA	NA	0.3	139.2
23	Patient	MCI	M	7	81	5	1	4	27	18	537	7248	0.074	+	531	100	+	0.186	NA	NA	10.2	42.6
24	Patient	MCI	F	10	74	3	9	15	35	20	2270	17090	0.133	-	818	81	+	0.036	NA	NA	1.6	89.8
25	Patient	MCI	M	20	73	0	11	12	32	11	1286	8397	0.153	-	190	36	-	0.028	NA	NA	2.3	42.9
26	Patient	MCI	F	12	65	0	17	21	31	4	1846	14207	0.130	-	397	54	-	0.029	NA	NA	3.4	31.8
27	Patient	MCI	F	13	78	10	11	13	27	3	730	6549	0.112	-	342	52	-	0.071	NA	NA	5.6	203.2
28	Patient	MCI	M	19	86	5	10	1	24	3	1260	7823	0.161	-	318	34	-	0.027	NA	NA	0.5	616.8
29	Patient	MCI	M	19	63	0	12	8	26	5	850	10503	0.081	+	699	106	+	0.125	NA	NA	1.3	71.8
30	Patient	MCI	M	15	61	1	10	13	29	9	1052	6262	0.168	-	340	35	-	0.033	NA	NA	8.7	188.6
31	Patient	MCI	M	16	64	0	13	4	26	0	1852	10190	0.182	-	358	29	-	0.016	NA	NA	3.7	819.1
32	Patient	MCI	F	13	60	6	15	14	35	13	1648	10191	0.162	-	385	41	-	0.025	NA	NA	32.2	1059.6
33	Patient	MCI	M	13	60	0	11	3	25	4	1003	11101	0.090	+	640	126	+	0.126	NA	NA	1.6	50.7
34	Patient	MCI	F	25	60	0	18	14	29	0	681	12364	0.055	+	778	125	+	0.183	NA	NA	0.1	44.6
35	Patient	MCI	M	13	71	1	15	2	26	0	486	NA	NA	+	80	8	-	0.016	NA	NA	4.5	92.2
36	Patient	Dem	F	6	86	0	7	25	34	27	407	NA	NA	+	160	15	-	0.036	NA	NA	5.6	57.5
37	Patient	Dem	M	18	70	2	14	2	28	4	470	NA	NA	+	156	13	-	0.029	NA	NA	3.4	75.1
38	Patient	MCI	F	16	53	1	11	1	35	8	2065	NA	NA	-	149	12	-	0.006	NA	NA	3.4	242.7
39	Patient	MCI	M	23	77	1	7	0	24	1	489	NA	NA	+	168	16	-	0.034	NA	NA	7.4	710.4
40	Patient	Dem	M	18	73	NA	NA	NA	NA	NA	445	NA	NA	+	351	46	+	0.104	NA	NA	6.1	1185.8
41	Patient	MCI	M	17	74	NA	9	24	35	14	581	NA	NA	+	167	16	-	0.027	NA	NA	3.2	58.4
42	Patient	MCI	M	20	71	1	NA	3	31	3	787	NA	NA	+	462	50	+	0.064	NA	+	7.4	165.1
43	Patient	MCI	F	11	71	1	10	6	32	8	801	NA	NA	+	406	40	+	0.049	NA	NA	5.6	126.2
44	Patient	MCI	M	NA	63	NA	14	NA	NA	NA	769	NA	NA	-	103	8	-	0.011	NA	NA	1.9	44.5
45	Patient	MCI	F	20	68	1	8	9	33	6	701	NA	NA	+	210	20	-	0.029	NA	NA	5.0	32.4
46	Patient	Dem	M	17	77	1	12	10	29	29	632	NA	NA	+	193	16	-	0.026	NA	NA	1.7	62.0
47	Patient	MCI	F	11	81	8	13	13	27	2	593	NA	NA	+	197	17	-	0.028	NA	NA	12.7	115.0
48	Patient	MCI	F	13	83	2	12	6	34	13	882	NA	NA	+	303	31	+	0.035	NA	+	18.1	106.1
49	Patient	MCI	F	NA	48	NA	NA	NA	NA	NA	1868	NA	NA	-	163	15	-	0.008	NA	NA	10.0	180.2
50	Patient	MCI	F	NA	66	NA	NA	NA	NA	NA	1868	NA	NA	-	252	18	-	0.010	NA	NA	31.7	45.7
51	Patient	MCI	F	18	68	0	13	3	35	2	776	NA	NA	+	197	20	-	0.026	NA	NA	21.9	35.3
52	Patient	MCI	M	18	63	0	12	0	30	0	437	NA	NA	+	90	8	-	0.018	NA	-	24.3	282.2

53	Patient	MCI	F	NA	83	NA	NA	NA	NA	NA	841	NA	NA	+	257	28	+	0.033	NA	NA	19.6	52.0
54	Patient	Dem	M	10	76	13	NA	26	33	23	721	NA	NA	+	287	33	+	0.045	NA	NA	10.5	127.9
55	Patient	Dem	F	16	56	1	NA	1	28	4	667	NA	NA	+	264	26	-	0.039	NA	NA	8.3	97.8
56	Patient	MCI	M	20	87	0	14	0	21	5	685	NA	NA	+	258	23	-	0.034	NA	NA	10.1	205.6
57	Patient	MCI	F	14	55	7	16	9	32	4	2415	NA	NA	-	173	15	-	0.006	NA	NA	11.6	48.4
58	Patient	MCI	F	NA	76	NA	NA	NA	NA	NA	980	NA	NA	-	164	19	-	0.019	NA	-	8.0	63.6
59	Patient	MCI	F	NA	79	6	9	13	26	2	949	NA	NA	+	299	28	+	0.029	NA	NA	5.5	214.9
60	Patient	MCI	M	16	59	8	12	30	29	13	1273	NA	NA	-	134	12	-	0.009	NA	NA	5.1	40.3
61	Patient	MCI	F	13	55	4	NA	36	34	3	851	NA	NA	-	95	8	-	0.009	NA	NA	9.3	95.0
62	Patient	MCI	F	11	72	2	17	1	28	0	673	NA	NA	+	199	16	-	0.024	NA	NA	5.7	49.3
63	Patient	MCI	F	19	73	0	14	0	23	2	710	NA	NA	+	386	30	+	0.043	NA	NA	27.7	332.5
64	Patient	MCI	M	14	63	2	9	17	25	2	550	NA	NA	+	358	34	+	0.062	NA	NA	20.9	91.0
65	Patient	Dem	M	19	65	1	6	24	32	27	517	NA	NA	+	152	15	-	0.029	+	NA	12.9	51.6
66	Patient	MCI	M	23	61	0	18	0	23	0	351	NA	NA	+	80	8	-	0.023	NA	NA	9.1	246.9
67	Patient	MCI	F	10	84	1	12	17	34	11	841	NA	NA	-	143	12	-	0.015	NA	NA	33.7	324.3
68	Patient	MCI	F	13	83	0	NA	NA	NA	NA	>2501	NA	NA	-	338	28	+	NA	NA	NA	7.7	190.2
69	Patient	MCI	F	13	74	3	15	11	27	5	753	NA	NA	+	515	61	+	0.081	NA	NA	6.8	92.5
70	Patient	MCI	F	9	69	3	3	42	26	4	683	NA	NA	+	208	19	-	0.028	NA	NA	1.9	134.6
71	Patient	Dem	F	NA	72	0	6	1	25	2	503	NA	NA	+	350	38	+	0.075	NA	NA	1.4	91.2
72	Patient	Dem	F	11	61	3	14	11	31	7	513	NA	NA	+	577	60	+	0.116	NA	+	3.2	53.4
73	Patient	MCI	F	20	58	2	18	13	28	2	1179	NA	NA	-	496	51	+	0.043	NA	+	5.9	78.4
74	Patient	Dem	F	13	65	0	7	12	35	10	419	NA	NA	+	293	27	+	0.065	NA	NA	3.1	26.8
75	Patient	MCI	F	NA	61	NA	NA	NA	NA	NA	1087	NA	NA	-	203	14	-	0.013	NA	NA	9.8	68.8
76	Patient	MCI	M	15	65	6	NA	NA	NA	NA	646	NA	NA	-	126	10	-	0.016	NA	NA	23.9	16.8
77	Patient	MCI	M	18	60	1	18	20	26	2	2318	NA	NA	-	221	18	-	0.008	NA	NA	10.7	220.1
78	Patient	MCI	F	11	83	0	16	7	25	3	991	NA	NA	+	395	37	+	0.037	NA	NA	23.0	84.8
79	Patient	MCI	F	11	55	13	15	34	31	8	1120	NA	NA	-	101	8	-	0.007	NA	NA	76.6	203.3
80	Patient	MCI	F	18	75	1	14	14	25	0	599	NA	NA	-	95	8	-	0.013	NA	NA	7.4	125.0
81	Patient	MCI	F	13	92	1	9	5	25	0	735	NA	NA	+	220	19	+	0.026	NA	+	3.6	153.4
82	Patient	MCI	M	11	76	1	17	8	27	0	807	NA	NA	+	375	39	+	0.048	NA	NA	6.5	209.7
83	Patient	MCI	M	12	64	3	12	14	30	2	375	NA	NA	+	165	14	-	0.037	NA	NA	2.9	37.2

84	Patient	SCD	F	15	79	7	18	9	29	0	1261	NA	NA	-	170	12	-	0.010	NA	NA	8.1	54.1
85	Patient	MCI	F	18	74	3	13	14	26	4	397	NA	NA	+	236	22	-	0.055	NA	NA	5.8	77.3
86	Patient	Dem	F	12	80	1	5	0	24	0	665	NA	NA	+	382	39	+	0.058	NA	NA	2.3	47.8
87	Patient	MCI	F	18	55	2	11	NA	NA	NA	709	NA	NA	+	386	41	+	0.058	NA	NA	12.7	96.5
88	Patient	MCI	M	17	75	5	5	16	27	18	471	NA	NA	+	359	33	+	0.070	NA	NA	14.9	46.9
89	Patient	Dem	F	13	65	1	4	3	26	4	679	NA	NA	+	236	21	-	0.031	NA	NA	1.1	89.2
90	Patient	MCI	M	17	67	2	13	0	21	0	415	NA	NA	+	137	14	-	0.034	NA	NA	11.3	58.9
91	Patient	MCI	F	14	71	5	13	12	30	1	856	NA	NA	+	412	41	+	0.048	NA	NA	1.1	46.5
92	Patient	MCI	M	16	67	2	14	15	29	1	1746	NA	NA	-	146	12	-	0.007	NA	NA	4.4	39.2
93	Patient	MCI	M	13	72	8	NA	22	34	15	1064	NA	NA	+	326	30	+	0.028	NA	NA	21.5	99.7
94	Patient	Dem	M	NA	80	NA	NA	NA	NA	NA	527	NA	NA	+	601	65	+	0.123	NA	NA	11.1	21.5
95	Patient	MCI	M	18	78	0	12	0	25	11	733	NA	NA	+	642	69	+	0.094	NA	NA	11.3	41.3
96	Patient	MCI	F	12	65	0	10	19	24	0	848	NA	NA	+	313	26	+	0.030	NA	NA	0.5	29.5
97	Patient	MCI	F	15	75	0	11	10	33	5	520	NA	NA	+	265	22	-	0.042	NA	NA	0.4	22.6
98	Patient	MCI	F	10	69	0	18	26	31	16	1030	NA	NA	-	124	9	-	0.009	NA	NA	17.8	57.5
99	Patient	MCI	M	21	76	0	15	4	27	3	682	NA	NA	+	164	12	-	0.018	+	NA	6.3	33.8
100	Patient	MCI	M	14	74	2	12	9	24	3	1934	NA	NA	-	199	14	-	0.007	NA	NA	7.6	76.9
101	Patient	MCI	M	14	74	1	9	17	32	19	421	NA	NA	+	243	19	-	0.045	NA	NA	10.6	121.7
102	HC	-	M	NA	21	9	15	0	0	24	NA	NA	NA	NA	NA	NA	NA	NA	NA	NA	0.1	10.9
103	HC	-	M	NA	18	3	15	8	0	11	NA	NA	NA	NA	NA	NA	NA	NA	NA	NA	2.0	24.8
104	HC	-	M	NA	62	0	14	0	0	21	NA	NA	NA	NA	NA	NA	NA	NA	NA	NA	0.1	34.5
105	HC	-	M	NA	70	7	10	4	0	22	NA	NA	NA	NA	NA	NA	NA	NA	NA	NA	0.1	18.4
106	HC	-	F	NA	58	0	17	6	0	21	NA	NA	NA	NA	NA	NA	NA	NA	NA	NA	0.2	27.4
107	HC	-	F	NA	74	4	12	7	3	10	NA	NA	NA	NA	NA	NA	NA	NA	NA	NA	1.1	66.9
108	HC	-	M	NA	70	0	12	3	3	23	NA	NA	NA	NA	NA	NA	NA	NA	NA	NA	0.1	98.9
109	HC	-	F	NA	55	1	18	1	0	19	NA	NA	NA	NA	NA	NA	NA	NA	NA	NA	6.5	241.4
110	HC	-	F	NA	20	4	15	5	1	17	NA	NA	NA	NA	NA	NA	NA	NA	NA	NA	0.1	73.8
111	HC	-	F	NA	20	5	16	12	2	19	NA	NA	NA	NA	NA	NA	NA	NA	NA	NA	5.9	160.5
112	HC	-	F	NA	78	4	18	5	0	25	NA	NA	NA	NA	NA	NA	NA	NA	NA	NA	0.8	120.4
113	HC	-	M	NA	46	1	18	7	0	22	NA	NA	NA	NA	NA	NA	NA	NA	NA	NA	0.2	71.5
114	HC	-	F	NA	66	0	17	4	0	19	NA	NA	NA	NA	NA	NA	NA	NA	NA	NA	1.3	657.5

115	HC	-	M	NA	75	0	14	16	0	28	NA	NA	NA	NA	NA	NA	NA	NA	NA	NA	2.7	378.6
116	HC	-	M	NA	74	2	13	2	0	23	NA	NA	NA	NA	NA	NA	NA	NA	NA	NA	0.1	25.5
117	HC	-	F	NA	70	0	18	10	1	21	NA	NA	NA	NA	NA	NA	NA	NA	NA	NA	2.7	64.0
118	HC	-	F	NA	63	4	18	0	0	21	NA	NA	NA	NA	NA	NA	NA	NA	NA	NA	3.4	63.3
119	HC	-	F	NA	66	7	18	2	0	26	NA	NA	NA	NA	NA	NA	NA	NA	NA	NA	1.9	46.6
120	HC	-	M	NA	34	7	17	6	0	12	NA	NA	NA	NA	NA	NA	NA	NA	NA	NA	1.7	58.6
121	HC	-	F	NA	72	1	18	6	0	27	NA	NA	NA	NA	NA	NA	NA	NA	NA	NA	1.6	56.8
122	HC	-	M	NA	77	5	15	2	0	26	NA	NA	NA	NA	NA	NA	NA	NA	NA	NA	0.8	27.0
123	HC	-	F	NA	60	0	18	6	0	26	NA	NA	NA	NA	NA	NA	NA	NA	NA	NA	0.4	64.3
124	HC	-	F	NA	49	1	17	24	0	28	NA	NA	NA	NA	NA	NA	NA	NA	NA	NA	6.5	51.1
125	HC	-	F	NA	64	2	18	6	0	24	NA	NA	NA	NA	NA	NA	NA	NA	NA	NA	0.5	72.9
126	HC	-	F	NA	61	2	17	3	0	22	NA	NA	NA	NA	NA	NA	NA	NA	NA	NA	0.2	1365.8
127	HC	-	M	NA	36	0	18	0	0	21	NA	NA	NA	NA	NA	NA	NA	NA	NA	NA	0.8	882.1
128	HC	-	M	NA	62	1	NA	3	0	21	NA	NA	NA	NA	NA	NA	NA	NA	NA	NA	0.2	104.6
129	HC	-	F	NA	60	0	18	7	0	25	NA	NA	NA	NA	NA	NA	NA	NA	NA	NA	0.1	28.5
130	HC	-	M	NA	52	0	14	0	0	23	NA	NA	NA	NA	NA	NA	NA	NA	NA	NA	0.2	98.9
131	HC	-	F	NA	66	2	15	3	0	24	NA	NA	NA	NA	NA	NA	NA	NA	NA	NA	0.1	32.0
132	HC	-	F	NA	63	4	17	11	0	13	NA	NA	NA	NA	NA	NA	NA	NA	NA	NA	1.3	27.3
133	HC	-	M	NA	59	1	NA	2	0	21	NA	NA	NA	NA	NA	NA	NA	NA	NA	NA	0.4	29.5
134	HC	-	F	NA	71	11	18	14	5	29	NA	NA	NA	NA	NA	NA	NA	NA	NA	NA	0.9	58.1
135	HC	-	F	NA	70	0	17	5	0	21	NA	NA	NA	NA	NA	NA	NA	NA	NA	NA	1.2	58.0
136	HC	-	F	NA	48	1	18	1	0	21	NA	NA	NA	NA	NA	NA	NA	NA	NA	NA	3.2	51.0
137	HC	-	M	NA	65	2	18	14	0	27	NA	NA	NA	NA	NA	NA	NA	NA	NA	NA	0.0	58.4
138	HC	-	M	NA	72	1	13	4	0	24	NA	NA	NA	NA	NA	NA	NA	NA	NA	NA	0.1	27.0
139	HC	-	F	NA	50	2	18	17	2	29	NA	NA	NA	NA	NA	NA	NA	NA	NA	NA	0.0	101.7
140	HC	-	M	NA	62	2	13	19	4	24	NA	NA	NA	NA	NA	NA	NA	NA	NA	NA	4.5	50.0
141	HC	-	M	NA	43	0	14	0	0	21	NA	NA	NA	NA	NA	NA	NA	NA	NA	NA	2.3	27.8
142	HC	-	M	NA	57	0	15	0	0	21	NA	NA	NA	NA	NA	NA	NA	NA	NA	NA	0.0	82.6
143	HC	-	F	NA	69	0	16	4	2	21	NA	NA	NA	NA	NA	NA	NA	NA	NA	NA	0.0	142.8
144	HC	-	F	NA	48	3	17	0	0	28	NA	NA	NA	NA	NA	NA	NA	NA	NA	NA	0.3	35.2
145	HC	-	F	NA	39	4	15	1	0	18	NA	NA	NA	NA	NA	NA	NA	NA	NA	NA	0.6	57.7

146	HC	-	F	NA	42	3	18	0	0	23	NA	NA	NA	NA	NA	NA	NA	NA	NA	NA	0.7	202.6
147	HC	-	F	NA	26	6	18	11	0	21	NA	NA	NA	NA	NA	NA	NA	NA	NA	NA	1.2	NA
148	HC	-	F	NA	53	1	17	4	0	25	NA	NA	NA	NA	NA	NA	NA	NA	NA	NA	18.7	92.1
149	HC	-	F	NA	24	6	17	12	0	23	NA	NA	NA	NA	NA	NA	NA	NA	NA	NA	27.9	507.6
150	HC	-	F	NA	52	4	13	32	0	21	NA	NA	NA	NA	NA	NA	NA	NA	NA	NA	23.2	20.8
151	HC	-	M	NA	20	8	18	26	0	24	NA	NA	NA	NA	NA	NA	NA	NA	NA	NA	1.7	10.8
152	HC	-	M	NA	78	0	13	5	0	27	NA	NA	NA	NA	NA	NA	NA	NA	NA	NA	23.9	21.7
153	HC	-	F	NA	54	4	16	26	7	27	NA	NA	NA	NA	NA	NA	NA	NA	NA	NA	35.9	18.7
154	HC	-	M	NA	26	0	18	0	0	21	NA	NA	NA	NA	NA	NA	NA	NA	NA	NA	26.1	6.2
155	HC	-	M	NA	38	3	17	6	0	23	NA	NA	NA	NA	NA	NA	NA	NA	NA	NA	8.5	5.5
156	HC	-	F	NA	81	4	10	8	5	27	NA	NA	NA	NA	NA	NA	NA	NA	NA	NA	10.1	20.9
157	HC	-	F	NA	61	5	18	12	0	28	NA	NA	NA	NA	NA	NA	NA	NA	NA	NA	14.6	16.1
158	HC	-	M	NA	64	3	13	1	0	24	NA	NA	NA	NA	NA	NA	NA	NA	NA	NA	16.9	20.8
159	HC	-	F	NA	52	NA	NA	NA	NA	NA	NA	NA	NA	NA	NA	NA	NA	NA	NA	NA	1.3	21.0
160	HC	-	F	NA	66	0	18	5	0	21	NA	NA	NA	NA	NA	NA	NA	NA	NA	NA	0.6	9.5
161	HC	-	F	NA	27	2	18	6	0	19	NA	NA	NA	NA	NA	NA	NA	NA	NA	NA	2.0	19.2
162	HC	-	M	NA	46	NA	NA	0	0	19	NA	NA	NA	NA	NA	NA	NA	NA	NA	NA	4.7	19.4
163	HC	-	M	NA	54	2	14	6	3	25	NA	NA	NA	NA	NA	NA	NA	NA	NA	NA	5.1	11.4
164	HC	-	F	NA	56	0	18	1	0	25	NA	NA	NA	NA	NA	NA	NA	NA	NA	NA	3.9	5.4
165	HC	-	M	NA	26	1	17	0	0	26	NA	NA	NA	NA	NA	NA	NA	NA	NA	NA	1.1	10.4
166	HC	-	F	NA	64	1	18	10	0	22	NA	NA	NA	NA	NA	NA	NA	NA	NA	NA	0.3	5.8
167	HC	-	M	NA	72	1	18	2	0	21	NA	NA	NA	NA	NA	NA	NA	NA	NA	NA	4.2	6.6
168	HC	-	F	NA	56	0	18	2	0	24	NA	NA	NA	NA	NA	NA	NA	NA	NA	NA	0.3	5.5
169	HC	-	F	NA	58	0	16	0	0	21	NA	NA	NA	NA	NA	NA	NA	NA	NA	NA	1.0	12.0
170	HC	-	M	NA	27	0	18	1	0	23	NA	NA	NA	NA	NA	NA	NA	NA	NA	NA	3.9	11.5
171	HC	-	M	NA	20	2	12	13	0	24	NA	NA	NA	NA	NA	NA	NA	NA	NA	NA	11.1	30.3
172	HC	-	F	NA	27	3	17	2	0	21	NA	NA	NA	NA	NA	NA	NA	NA	NA	NA	44.8	68.3
173	HC	-	F	NA	73	0	18	0	0	21	NA	NA	NA	NA	NA	NA	NA	NA	NA	NA	5.3	9.9
174	HC	-	F	NA	73	NA	NA	NA	NA	NA	NA	NA	NA	NA	NA	NA	NA	NA	NA	NA	2.4	23.5
175	HC	-	F	NA	62	7	18	12	0	26	NA	NA	NA	NA	NA	NA	NA	NA	NA	NA	3.3	25.7
176	HC	-	F	NA	80	0	17	1	0	23	NA	NA	NA	NA	NA	NA	NA	NA	NA	NA	1.5	11.9

177	HC	-	M	NA	47	0	18	1	0	22	NA	NA	NA	NA	NA	NA	NA	NA	NA	NA	9.5	25.5
178	HC	-	M	NA	57	1	18	3	0	21	NA	NA	NA	NA	NA	NA	NA	NA	NA	NA	4.1	11.0
179	HC	-	M	NA	83	0	17	0	0	21	NA	NA	NA	NA	NA	NA	NA	NA	NA	NA	0.8	8.2
180	HC	-	M	NA	48	1	11	2	1	24	NA	NA	NA	NA	NA	NA	NA	NA	NA	NA	3.6	13.4
181	HC	-	F	NA	21	0	18	3	1	25	NA	NA	NA	NA	NA	NA	NA	NA	NA	NA	1.8	13.3
182	HC	-	M	NA	34	3	18	1	0	21	NA	NA	NA	NA	NA	NA	NA	NA	NA	NA	18.6	23.0
183	HC	-	F	NA	26	0	16	5	21	0	NA	NA	NA	NA	NA	NA	NA	NA	NA	NA	7.6	8.4
184	HC	-	F	NA	60	5	18	16	0	28	NA	NA	NA	NA	NA	NA	NA	NA	NA	NA	14.4	164.7
185	HC	-	M	NA	30	3	18	4	0	27	NA	NA	NA	NA	NA	NA	NA	NA	NA	NA	5.6	22.8
186	HC	-	M	NA	31	9	12	10	0	21	NA	NA	NA	NA	NA	NA	NA	NA	NA	NA	7.9	14.8
187	HC	-	F	NA	39	0	10	20	1	25	NA	NA	NA	NA	NA	NA	NA	NA	NA	NA	1.2	98.9
188	HC	-	F	NA	41	0	17	4	0	26	NA	NA	NA	NA	NA	NA	NA	NA	NA	NA	16.1	23.4
189	HC	-	M	NA	18	1	18	0	0	21	NA	NA	NA	NA	NA	NA	NA	NA	NA	NA	8.9	5.6
190	HC	-	F	NA	36	3	16	22	1	19	NA	NA	NA	NA	NA	NA	NA	NA	NA	NA	0.3	4.4
191	HC	-	F	NA	79	NA	NA	NA	NA	NA	NA	NA	NA	NA	NA	NA	NA	NA	NA	NA	4.8	5.9
192	HC	-	F	NA	72	0	18	11	0	26	NA	NA	NA	NA	NA	NA	NA	NA	NA	NA	4.3	20.7
193	HC	-	M	NA	68	7	14	5	0	24	NA	NA	NA	NA	NA	NA	NA	NA	NA	NA	7.0	17.6
194	HC	-	M	NA	60	3	17	6	0	21	NA	NA	NA	NA	NA	NA	NA	NA	NA	NA	2.6	4.9
195	HC	-	F	NA	53	0	18	6	0	24	NA	NA	NA	NA	NA	NA	NA	NA	NA	NA	2.6	11.7
196	HC	-	M	NA	33	0	18	9	0	21	NA	NA	NA	NA	NA	NA	NA	NA	NA	NA	0.4	7.3
197	HC	-	F	NA	42	3	18	22	1	24	NA	NA	NA	NA	NA	NA	NA	NA	NA	NA	2.1	30.2
198	HC	-	F	NA	79	0	18	0	1	27	NA	NA	NA	NA	NA	NA	NA	NA	NA	NA	8.6	4.1
199	HC	-	F	NA	46	24	18	34	0	25	NA	NA	NA	NA	NA	NA	NA	NA	NA	NA	10.8	38.1
200	HC	-	F	NA	28	4	18	15	0	21	NA	NA	NA	NA	NA	NA	NA	NA	NA	NA	3.8	18.2
201	HC	-	F	NA	69	1	18	9	1	25	NA	NA	NA	NA	NA	NA	NA	NA	NA	NA	2.0	4.9
202	HC	-	F	NA	65	2	18	0	0	25	NA	NA	NA	NA	NA	NA	NA	NA	NA	NA	9.5	6.6
203	HC	-	M	NA	68	4	18	2	0	24	NA	NA	NA	NA	NA	NA	NA	NA	NA	NA	2.0	14.9
204	HC	-	F	NA	69	8	17	14	0	17	NA	NA	NA	NA	NA	NA	NA	NA	NA	NA	3.1	27.1
205	HC	-	F	NA	27	1	18	1	0	23	NA	NA	NA	NA	NA	NA	NA	NA	NA	NA	9.8	13.5
206	HC	-	F	NA	57	NA	NA	NA	NA	NA	NA	NA	NA	NA	NA	NA	NA	NA	NA	NA	7.5	3.5

Abbreviations: Aβ, amyloid-beta; BDI II, Beck Depression Inventory-II; CCCSS, Cleveland Clinic Constipation Scoring System; Dem, dementia; FAQ, Functional Activities Questionnaire; HC, healthy control; MAC-Q, Memory Assessment Clinic-Questionnaire; MCI, mild cognitive impairment; NA, not available; PET, positron emission tomography; SCD, subjective cognitive decline; +, positive; -, negative.
