# Supplementary material for: Fecal Aβ and tau aggregates are elevated in cognitive impairment and reveal peripheral proteopathic alterations
Source: Alzheimers Res Ther. 2026 Sep 26;18:215. doi: 10.1186/s13195-026-02188-0 (PMC13621666; doi:10.1186/s13195-026-02188-0)
Supplement: Supplementary file 3 [file 13195_2026_2188_MOESM3_ESM.pdf]

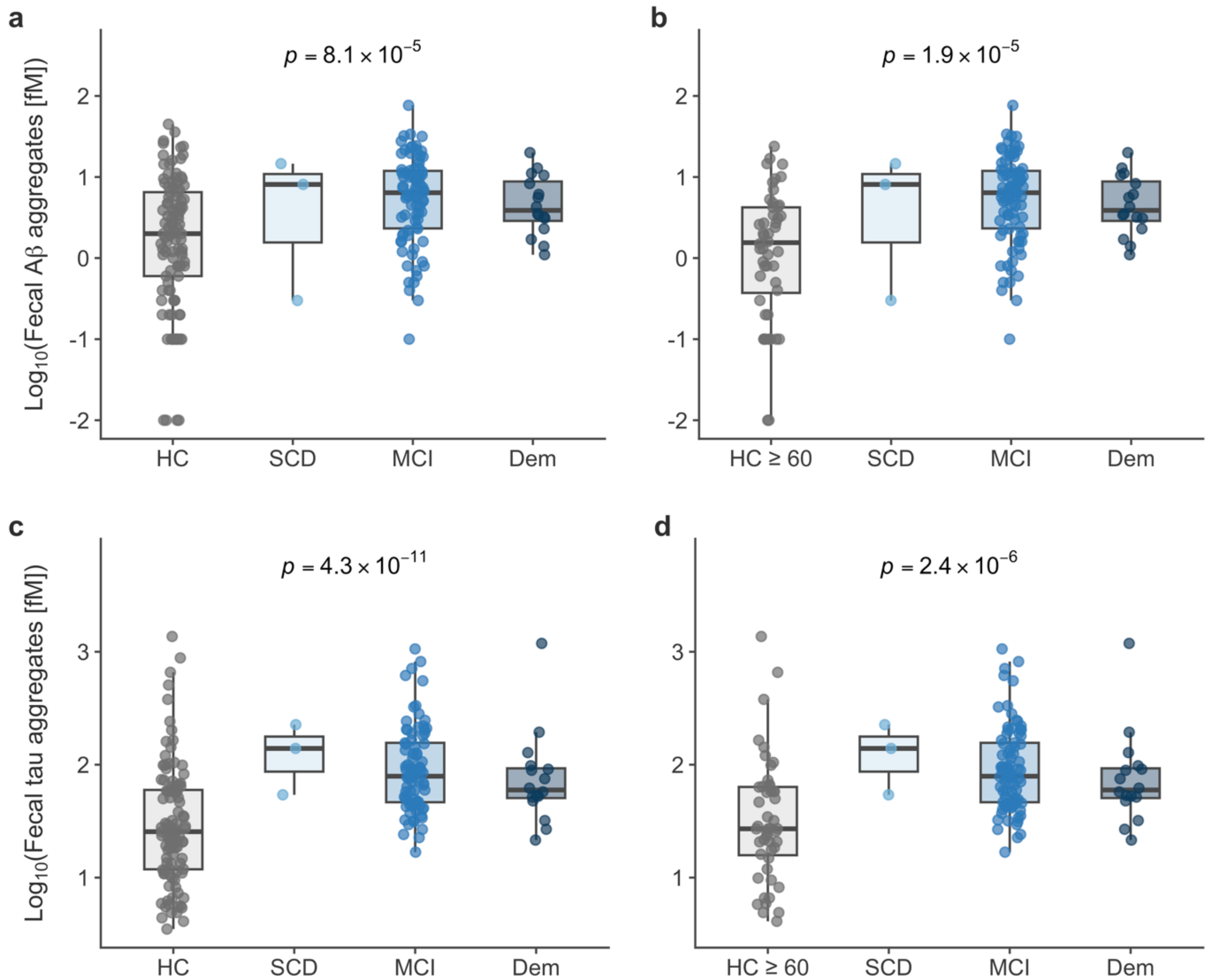

Figure S2 Fecal Aβ and tau aggregates remain elevated across clinical stages of cognitive impairment. **a** Fecal Aβ aggregates in healthy controls (HC), subjective cognitive decline (SCD), mild cognitive impairment (MCI), and dementia (Dem) across all participants. **b** Same analysis restricted to age-matched controls (HC ≥ 60 years). **c** Fecal tau aggregates across all diagnostic groups. **d** Same analysis restricted to HC ≥ 60 years. Data are shown as box plots (median and interquartile range; whiskers indicate Tukey limits) with individual observations. Statistical significance was assessed using Kruskal–Wallis tests; *p*-values are shown in the figure. Exploratory pairwise Mann–Whitney U tests showed no significant differences among the SCD, MCI, and dementia groups for either fecal Aβ or fecal tau aggregates. Compared with HC, MCI

and dementia showed elevated fecal Aβ and tau aggregate concentrations; comparisons involving SCD should be interpreted cautiously because of the small subgroup size.
