# Supplementary material for: Fecal Aβ and tau aggregates are elevated in cognitive impairment and reveal peripheral proteopathic alterations
Source: Alzheimers Res Ther. 2026 Sep 26;18:215. doi: 10.1186/s13195-026-02188-0 (PMC13621666; doi:10.1186/s13195-026-02188-0)
Supplement: Supplementary file 4 [file 13195_2026_2188_MOESM4_ESM.pdf]

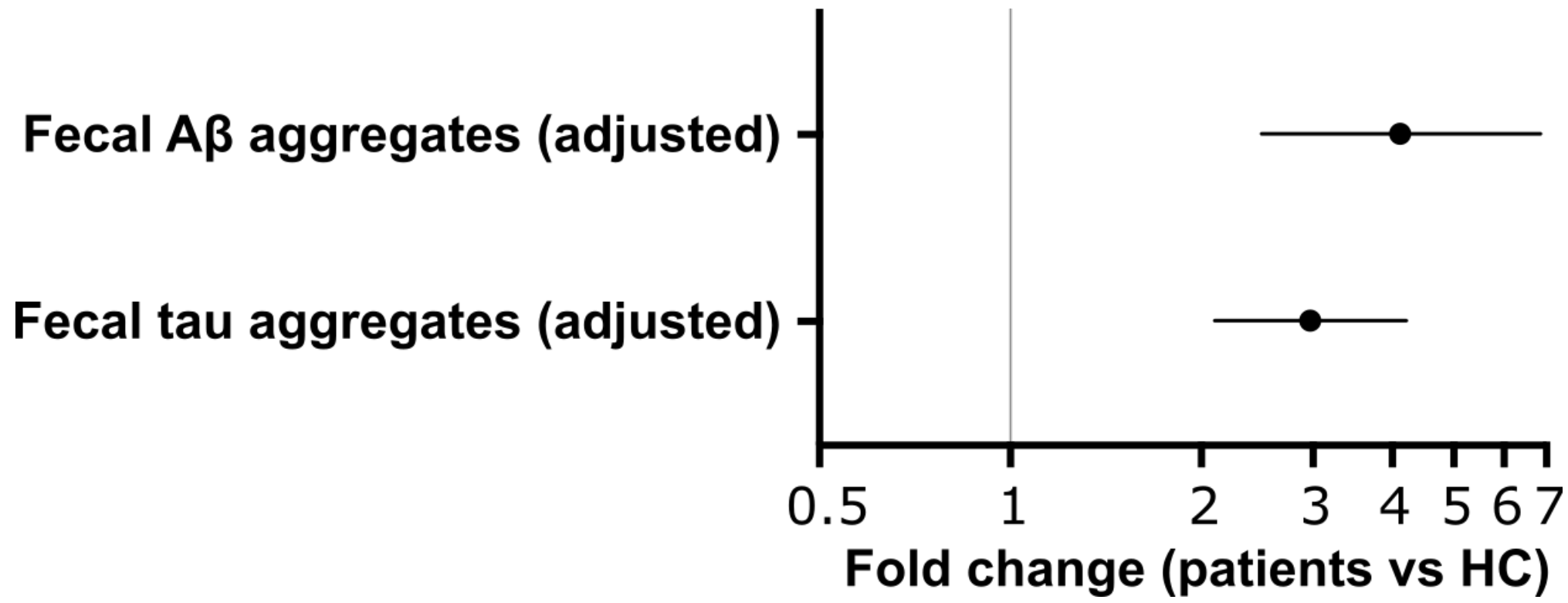

Figure S3 Demographic-adjusted associations between cognitive impairment and fecal aggregate concentrations. Forest plot showing adjusted fold-change estimates for fecal Aβ and fecal tau aggregate concentrations in patients with cognitive impairment relative to cognitively healthy controls. Estimates were derived from multivariable linear regression models of $\log_{10}$-transformed concentrations including age and sex as covariates. Points indicate fold-change estimates and horizontal lines indicate 95% confidence intervals. The vertical reference line denotes no difference (fold-change = 1).
