# Supplementary material for: Fecal Aβ and tau aggregates are elevated in cognitive impairment and reveal peripheral proteopathic alterations
Source: Alzheimers Res Ther. 2026 Sep 26;18:215. doi: 10.1186/s13195-026-02188-0 (PMC13621666; doi:10.1186/s13195-026-02188-0)
Supplement: Supplementary file 5 [file 13195_2026_2188_MOESM5_ESM.pdf]

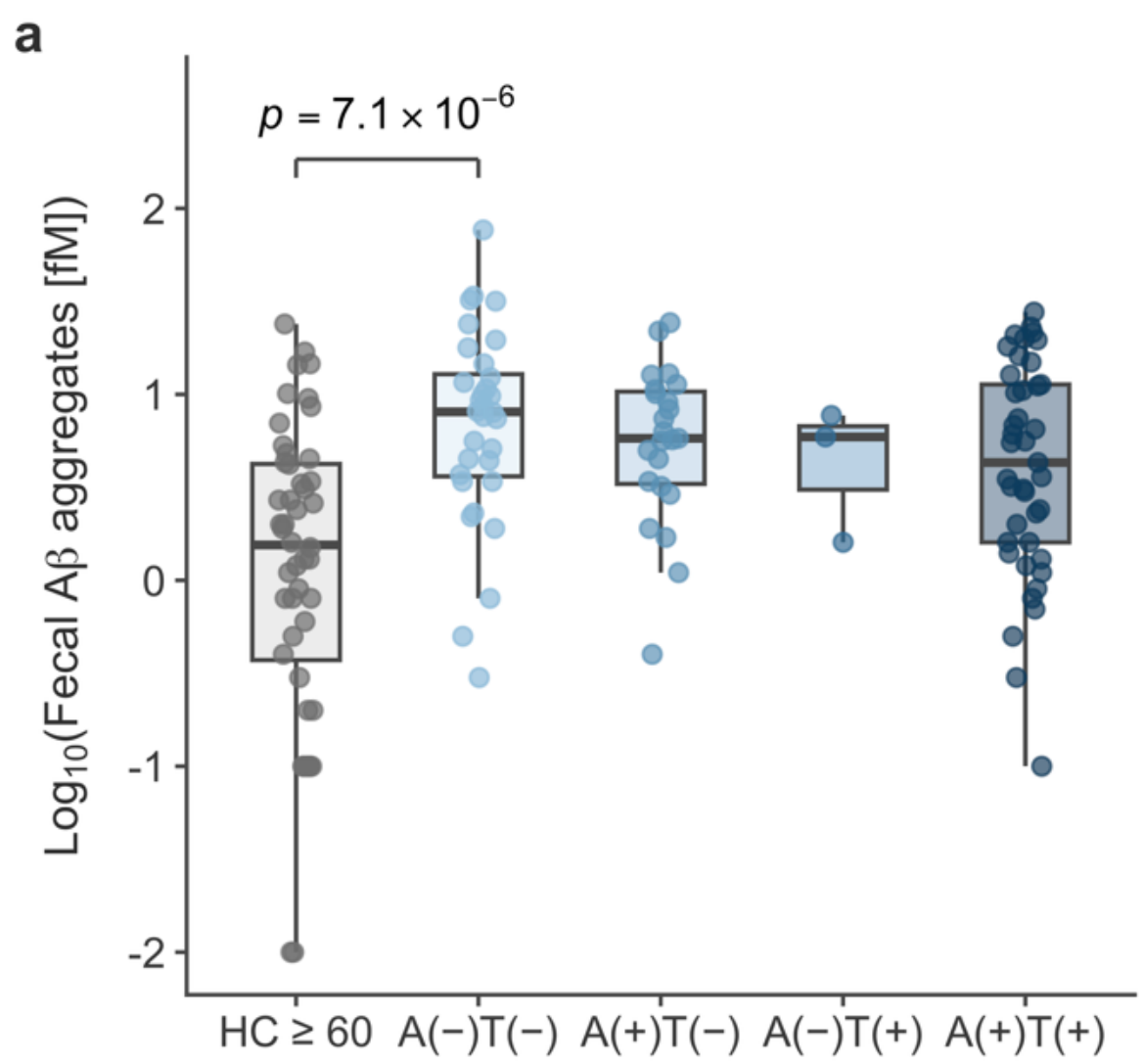

Figure S4 Fecal Aβ and tau aggregate elevations across AT-defined strata persist after restriction to controls aged ≥60 years. **a**, **b** Log_{10}-transformed fecal Aβ (**a**) and tau (**b**) aggregate concentrations across age-matched cognitively healthy controls (HC ≥ 60 years) and CSF-defined AT biomarker categories. Elevated fecal aggregate levels are observed in patients across AT strata, including biomarker-negative individuals (A(−)T(−)), indicating that the association between fecal aggregate burden and cognitive impairment is not explained by age differences. Data are shown as box plots (median and interquartile range; whiskers indicate Tukey limits) with individual observations. Annotated p-values indicate planned pairwise comparisons between HC ≥ 60 and the A(−)T(−) subgroup using two-sided Mann–Whitney U tests.
